# Exploring the role of Chinese herbal medicine in the long-term management of postoperative ovarian endometriotic cysts: a systematic review and meta-analysis

**DOI:** 10.3389/fphar.2024.1376037

**Published:** 2024-06-07

**Authors:** Danni Ding, Shaoxuan Liu, Fangyuan Liu, Songli Hao, Chunlan Zhang, Ying Shen, Wei Wei, Qiaochu Chen, Fengjuan Han

**Affiliations:** ^1^ Heilongjiang University of Chinese Medicine, First Clinical Medical College, Harbin, China; ^2^ The First Affiliated Hospital of Heilongjiang University of Chinese Medicine, Harbin, China

**Keywords:** Chinese herbal medicine, ovarian endometriotic cyst, postoperative treatment, randomized controlled trial, systematic review, meta-analysis

## Abstract

**Background:**

Ovarian endometriotic cysts (OEC) represent the primary manifestation of endometriosis, constituting a hormonally dependent inflammatory disorder in gynecology. It significantly affects the quality of life and reproductive health of women. It is worth noting that traditional Chinese medicine (TCM), especially Chinese herbal medicine (CHM), has been widely applied in mainland China due to its unique therapeutic system and commendable clinical efficacy, bringing new hope for preventing and managing OEC.

**Objective:**

This study aims to evaluate the efficacy and safety of CHM in the management of postoperative OEC. Simultaneously, it seeks to explore the medication laws, therapeutic principles, and specific treatment mechanisms of CHM.

**Methods:**

Eight electronic databases were searched from their inception to 01 November 2023. Randomized controlled trials (RCTs) assessing the therapeutic effects and safety of CHM for postoperative OEC were included. The risk of bias for each trial was assessed using the Cochrane Collaboration’s tool. The certainty of the evidence was evaluated using the GRADE profiler 3.2. Additionally, we extracted formulation from the included studies, conducting a thorough analysis.

**Results:**

**(ⅰ)** Twenty-two RCTs involving 1938 patients were included. In terms of the primary efficacy outcome, the CHM group demonstrated a potentially lower recurrence rate compared to both control (odds ratio (OR) = 0.25; 95% confidence intervals (CI): 0.10–0.64) and conventional western medicine (CWM) (OR = 0.26; 95% CI: 0.11–0.65) groups. Furthermore, the joint application of CHM and CWM resulted in a significant reduction in the recurrence rate (OR = 0.26; 95% CI: 0.17–0.40). **(ⅱ)** Regarding secondary efficacy outcomes, (a) Total clinical efficacy rate: CHM showcased an augmentation in clinical effectiveness compared to both the control (OR = 4.23; 95% CI: 1.12–15.99) and CWM (OR = 2.94; 95% CI: 1.34–6.43) groups. The combined administration of CHM and CWM substantially enhanced overall clinical effectiveness (OR = 3.44; 95% CI: 2.37–5.00). (b) VAS Score: CHM exhibited the capacity to diminish the VAS score in comparison to surgery alone (Mean difference (MD) = −0.86; 95% CI: -1.01 to −0.71). Nevertheless, no substantial advantage was observed compared to CWM alone (MD = −0.16; 95% CI: -0.49 to 0.17). The integration of CHM with CWM effectively ameliorated pain symptoms (MD = −0.87; 95% CI: -1.10 to −0.65). (c) Serum Level of Cancer antigen 125 (CA125): the CHM group potentially exhibited lower CA125 levels in comparison to CWM alone (MD = −11.08; 95% CI: -21.75 to −0.42). The combined intervention of CHM and CWM significantly decreased CA125 levels (MD = −5.31; 95% CI: -7.27 to −3.36). (d) Pregnancy Rate: CHM exhibited superiority in enhancing the pregnancy rate compared to surgery (OR = 3.95; 95% CI: 1.60–9.74) or CWM alone (OR = 3.31; 95% CI: 1.40–7.83). The combined utilization of CHM and CWM demonstrated the potential to enhance pregnancy rates compared to CWM (OR = 2.99; 95% CI: 1.28–6.98). Concerning safety outcome indicators, CHM effectively decreased the overall incidence of adverse events and, to a certain extent, alleviated perimenopausal symptoms as well as liver function impairment. **(ⅲ)** Most of CHMs were originated from classical Chinese herbal formulas. *Prunus persica* (L.) Batsch (Taoren), *Angelica sinensis* (Oliv.) Diels (Danggui), *Salvia miltiorrhiza* Bunge (Danshen), *Paeonia lactiflora* Pall. (Chishao), and *Corydalis yanhusuo* W.T.Wang (Yanhusuo) were most frequently used CHM.

**Conclusion:**

CHM may be a viable choice in the long-term management of postoperative OEC, with the potential to enhance clinical efficacy while decreasing recurrence and adverse effects.

## 1 Introduction

Ovarian endometriotic cysts (OEC) arise from the growth of ectopic endometrial tissue within the ovarian cortex (Bulun et al., 2019), constituting a major manifestation of endometriosis and contributing to 17%–44% of its incidence (Wang G. et al., 2022). Clinically, OEC is characterized by pelvic pain, infertility, abnormal menstruation, significantly impacting the quality of life and reproductive health of women of childbearing age (Bonavina and Taylor, 2022). Currently, conservative surgery is the principal treatment for reproductive-age patients with OEC (Falcone and Flyckt, 2018). Unfortunately, the recurrence rate within 5 years post-surgery can reach up to 50% (Ceccaroni et al., 2019), rendering OEC a chronic condition. Post-surgery, dienogest and gonadotropin-releasing hormone agonists (GnRH-α) are commonly prescribed to eradicate microscopic lesions and mitigate OEC recurrence. Yet, prolonged use may suppress ovarian function and delay pregnancy (Zhao, 2023). Furthermore, adverse effects including vasoconstriction symptoms, insomnia, irregular vaginal bleeding, and gastrointestinal discomfort, pose significant challenges in the long-term management post-OEC surgery (Sauerbrun-Cutler and Alvero, 2019; Della Corte et al., 2020; Konninckx et al., 2021). According to a report in The Lancet (Taylor et al., 2021), endometriosis has been considered a systemic disease that may affect liver and adipose tissue metabolism, trigger systemic inflammation, alter brain gene expression, and lead to pain sensitization and mood disorders (Taylor et al., 2021). Therefore, seeking more effective and tolerable postoperative treatments for OEC holds significant clinical relevance.

In recent years, Chinese herbal medicine (CHM) has emerged as a promising alternative therapy within the field of gynecology due to its unique treatment system. Contemporary research has further validated the crucial role of CHM in treating a variety of gynecological diseases, including OEC. For instance, studies have shown CHM’s ability to induce apoptosis in ovarian cancer cells through multiple signaling pathways (Wang et al., 2024), ameliorate perimenopausal syndrome by regulating hormones secreted by the ovaries (Xue et al., 2022), and exhibit potential therapeutic effects on pregnancy-related diseases, such as recurrent spontaneous abortion, pre-eclampsia, and gestational diabetes (Fang et al., 2023). Furthermore, a meta-analysis demonstrated that CHM significantly alleviates pain associated with endometriosis with fewer side effects compared to conventional therapies (Lin et al., 2023). These findings affirm the applicability of CHM in treating gynecological diseases and provide a scientific basis for its integration into the modern medical system. Therefore, exploring the role of CHM in the long-term management of postoperative OEC is not only a venture into the modern application of traditional medicine but also critically important for the advancement and development of innovative treatment methods in gynecology.

In the postoperative treatment of OEC, CHM adopts a patient-centered approach, adhering to natural law principles and emphasizing holistic care. It aims to bolster healthy qi, dispel pathogen, and facilitate the body’s recovery (Xu et al., 2020). The mechanisms through which CHM aids in the postoperative management of OEC primarily include: 1) regulating the immune system to promote immune balance and enhance immunity, thereby facilitating the elimination of ectopic endometrial tissue (Zhang et al., 2013; Shen et al., 2016; Song et al., 2021); 2) exerting anti-inflammatory effects to significantly reduce postoperative inflammation and alleviate pain (MERESMAN et al., 2021; Chen et al., 2024); 3) modulating the endocrine system to correct endocrine dysregulation, inhibit the proliferation of ectopic tissue, and restore ovarian function (Lai et al., 2021; Ma et al., 2021; Zhao, 2023); (4) promoting blood circulation to repair tissue damage and decrease the recurrence of OEC (Zhou et al., 2019; Zhao et al., 2020); (5) directly targeting ectopic endometrial tissue to inhibit its growth and invasion (Pang et al., 2021; Tan et al., 2021; Huang et al., 2022; Zhang et al., 2023). Collectively, CHM plays a comprehensive and multi-level therapeutic role in the long-term management of postoperative OEC.

A considerable volume of clinical research, encompassing case reports, case series, and randomized controlled trials (RCTs), has demonstrated that CHM could decrease recurrence rates, boost pregnancy rates, and improve the quality of life, bringing new hope for clinical management and the development of novel therapeutic approaches for postoperative OEC (Ma, 2011; Ding and Shi, 2012; Zhang, 2012; Zhou and Liu, 2013; Li, 2014; Chen, 2015; Chen et al., 2015; Dou, 2015; Du, 2015; Han, 2016; Xing, 2016; Zhou, 2016; Chen, 2018; Hu and Y, 2018; Lu et al., 2019; Qiu and Wan, 2019; Song et al., 2019; Wang and Bo, 2019; Liu, 2020; Zhao and Xiao, 2020; Wu and Deng, 2021; Tong et al., 2023). Despite this, debates regarding its efficacy and safety persist. Although 3 systematic reviews regarding CHM treatment for postoperative OEC have been published in advance respectively, some issues were also identified. Two reviews investigated the efficacy of *Salvia miltiorrhiza*-containing CHM (Gao Q. et al., 2022) and *black cohosh* extracts (Peng et al., 2020) in improving the low estrogen status induced by GnRH-α in postoperative endometriosis patients. However, the outcome measures and the scope of exploring CHM were relatively limited. Regarding the review by Dr. Fan et al., (Fan et al., 2022), more databases and RCTs should be updated to reduce potential bias. Therefore, this study has updated the relevant literature, conducted a comprehensive systematic review of RCTs to evaluate the current clinical evidence on CHM for postoperative OEC. Additionally, it provided a summarizing analysis of medication characteristics and treatment principles, aiming to offer assistance for clinical medication.

## 2 Methods

This study was conducted and reported according to the guidelines of Preferred Reporting Items for Systematic Reviews and Meta-Analyses (PRISMA) 2020 (Page et al., 2021) (Supplementary Appendix S1).

### 2.1 Eligibility criteria

#### 2.1.1 Types of studies

RCTs which have evaluated the efficacy of CHM for OEC were included in this study.

#### 2.1.2 Types of participants

Patients received conservative surgical intervention (laparoscopic or laparotomy excision or ablation of lesions while preserving the uterus and ovaries) and had a pathological diagnosis of OEC. In order to ensure including all relevant studies, no restriction on age and nationality was specified.

#### 2.1.3 Types of interventions

Patients in the treatment group should be treated by CHM or combination of CHM and CWM. Patients in the control group should be treated by CWM or CHM placebo or be a blank control. CWM in the treatment and control group must be identical in name, usage, dosage, etc. No restrictions on dosage forms, route of administration, quantity, or treatment course of CHM was specified.

#### 2.1.4 Types of outcome measures

The primary efficacy outcome measure was defined as recurrence rates, while secondary outcomes consist of total clinical efficacy rate, VAS score, serum level of Cancer antigen 125 (CA125), and pregnancy rate. Safety outcome measures encompass the total incidence of adverse events as well as the specific rate of adverse reactions.

#### 2.1.5 Exclusion criteria

RCTs will be excluded if the following conditions are met: (a) clinical experiences, theoretical discussion, reviews, commentaries, editorials, case reports, case series, and experimental studies; (b) Studies limited to surgical exploration, diagnosis, or staging without any intervention on the lesions in patients; (c) Control groups receiving CHM treatment, with either no established control group or inconsistency in baseline treatments between groups; (d) no detailed information regarding clinical efficacy could be extracted; and (e) duplicate publications.

### 2.2 Literature search

Relevant literature assessing the efficacy and safety of CHM for OEC was searched in 8 electronic databases including PubMed, Web of Science, Cochrane Library, EMBASE, Chinese National Knowledge Infrastructure (CNKI), VIP Information Database (VIP), Chinese Biomedical Literature Database (CBM), and Wanfang Database from inception up to 01 November 2023. The following grouped keywords and Mesh/Emtree thesauri were used as search terms and modified according to each database: “Ovarian endometriotic cyst” “ovarian chocolate cyst” “Postoperative Period” “Surgical Procedures, Operative” “Laparoscopes” “Chinese herbal medicine” “traditional Chinese medicine” “zhong yi yao” “zhong yao” “formula” “decoction” “pill” “capsule” “granules” “powder” “paste” “recipe” “clinical trial” “randomized controlled trial”, etc. Two different authors (DN Ding and SX Liu) independently conducted the literature search and evaluated the results. The search strategies for all the electronic databases can be found in Supplementary Appendix S2. To minimize bias, we also retrieved the ongoing registered clinical trials and unpublished papers on CHM for OEC. No language and status restriction were set in this review.

### 2.3 Study selection and data extraction

Trials were selected according to the inclusion/exclusion criteria by reading the titles, abstracts and (or) full texts of the published articles. Two authors (SX Liu and FY Liu) independently selected the studies and extracted data using a pre-designed data extraction sheet, evaluated and cross-checked. Detailed information of enrolled study was listed as follows: (a) basic characteristics of included studies: title of study, authors’ name, publication date, sample size, diagnostic criteria, methodological quality, therapeutic schedule in treatment and control groups, components and dosage of CHM, withdrawals, and course of treatment; (b) basic characteristics of included patients: age, gender, duration of disease, size and staging of OEC, previous medical history, and laboratory examination; (c) both primary and secondary outcome measures; and (d) adverse effects. If disagreements on data extraction were identified, a third party (FJ Han) was consulted.

### 2.4 Assessment of methodological quality

Methodological quality of the included trials was also assessed by 2 authors (SL Hao and Y Shen) independently. According to Cochrane Collaboration’s tool (Sterne et al., 2019), 7 fields of risk of bias (ROB) were evaluated as below: random sequence generation, allocation concealment, blinding of participants and personnel, blinding of outcome assessment, incomplete outcome data, selective reporting, and other bias. Each field was assessed to be “yes” (low ROB), “no” (high ROB), or “unclear” (unclear ROB). The inconsistencies were discussed with the third author (FJ Han).

### 2.5 Data analysis

Review Manager software (RevMan, Version 5.4, Copenhagen: The Nordic Cochrane Centre, The Cochrane Collaboration, 2020) was utilized to conduct data analysis of dichotomous and continuous outcome measures, which were extracted from the original studies. Mean difference (MD) was utilized for data measurement of continuous outcomes, while odds ratio (OR) for dichotomous outcomes. All of them were expressed with a 95% confidence interval (CI). When no statistical heterogeneity was identified (heterogeneity test, *p* ≥ 0.10, or *I* ^*2*^ ≤ 50%), fixed-effects model was selected, otherwise random-effects model was applied. Subgroup analysis and/or sensitivity analysis was conducted to identify the sources of heterogeneity. Funnel plot was also used to evaluate the publication bias when over 10 trials were included in the analysis. A significant difference was considered when *p* < 0.05.

Furthermore, in accordance with the GRADE (Grading of Recommendations Assessment, Development and Evaluation criteria) (Guyatt et al., 2008a), the quality of evidence for each outcome was assessed using GRADE profiler 3.2.

## 3 Results

### 3.1 Study selection

**FIGURE 1:**
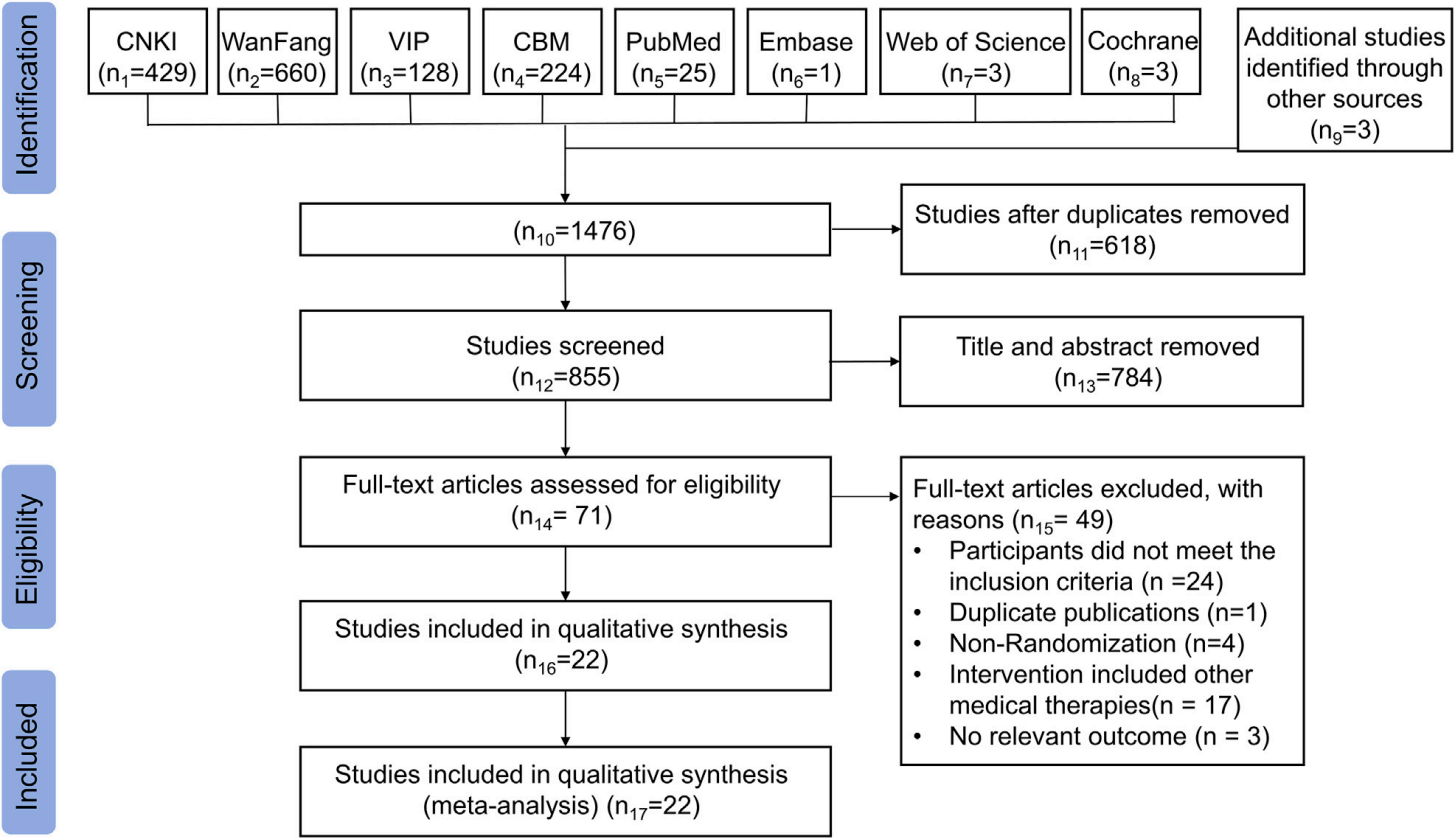
Flow diagram of study selection and identification.

The flowchart of literature identification and screening is depicted in Figure 1. In total, 1,476 related literature was derived from the above 8 electronic databases, among which three are ongoing clinical trials (Chictr, 2020; Chictr, 2023). After removing duplicate publications, 855 studies remained. Subsequently, 784 studies were excluded for not being RCTs, specifically including reviews, commentaries, editorials, case reports, experimental studies, data mining articles, and irrelevant to postoperative OEC after scanning titles and abstracts. Furthermore, after reviewing the remaining 71 full texts, an additional 49 studies were excluded for the following reasons: participants did not meet the inclusion criteria (n = 24); duplicate publications (n = 1); non-randomization (n = 4); intervention included other medical therapies (n = 17); no relevant outcome (n = 3). Ultimately, 22 eligible RCTs were included (Ma, 2011; Ding and Shi, 2012; Zhang, 2012; Zhou and Liu, 2013; Li, 2014; Chen, 2015; Chen et al., 2015; Dou, 2015; Du, 2015; Han, 2016; Xing, 2016; Zhou, 2016; Chen, 2018; Hu and Y, 2018; Lu et al., 2019; Qiu and Wan, 2019; Song et al., 2019; Wang and Bo, 2019; Liu, 2020; Zhao and Xiao, 2020; Wu and Deng, 2021; Tong et al., 2023).

### 3.2 Characteristics of included trials

The basic characteristics of enrolled studies and subjects are presented in Table 1, including sample size, age, intervention, treatment duration, follow-up duration, and outcome measures. All included studies were conducted in China and published in Chinese between 2011 and 2023. There were altogether 1938 patients enrolled in this review, with the sample sizes ranged from 48 to 160. Four studies assessed the efficacy of CHM alone compared to CWM, one study compared the efficacy of CHM alone with a blank control, and the remaining studies evaluated the efficacy of CHM combined with CWM compared to standalone CWM. Treatment duration varied from 3 to 6 months. Mean follow-up durations ranged from 3 to 36 months. Recurrence rate was reported in 17 studies (Ma, 2011; Zhang, 2012; Zhou and Liu, 2013; Li, 2014; Chen, 2015; Chen et al., 2015; Dou, 2015; Han, 2016; Xing, 2016; Zhou, 2016; Hu and Y, 2018; Qiu and Wan, 2019; Song et al., 2019; Wang and Bo, 2019; Liu, 2020; Zhao and Xiao, 2020; Tong et al., 2023). Total clinical efficacy rates were reported in 16 trials (Ma, 2011; Ding and Shi, 2012; Zhou and Liu, 2013; Li, 2014; Chen et al., 2015; Dou, 2015; Du, 2015; Zhou, 2016; Chen, 2018; Hu and Y, 2018; Lu et al., 2019; Song et al., 2019; Wang and Bo, 2019; Zhao and Xiao, 2020; Wu and Deng, 2021; Tong et al., 2023). VAS score was reported in 10 trials (Zhang, 2012; Chen, 2015; Chen et al., 2015; Han, 2016; Xing, 2016; Hu and Y, 2018; Song et al., 2019; Zhao and Xiao, 2020; Wu and Deng, 2021; Tong et al., 2023). Serum level of CA125 was reported in 11 trials (Zhang, 2012; Chen, 2015; Dou, 2015; Du, 2015; Han, 2016; Xing, 2016; Zhou, 2016; Hu and Y, 2018; Lu et al., 2019; Liu, 2020; Zhao and Xiao, 2020). Pregnancy rate was reported in 6 trials (Ding and Shi, 2012; Li, 2014; Qiu and Wan, 2019; Liu, 2020; Zhao and Xiao, 2020; Tong et al., 2023). Fourteen trials reported adverse events (Ding and Shi, 2012; Zhang, 2012; Zhou and Liu, 2013; Chen, 2015; Dou, 2015; Du, 2015; Han, 2016; Xing, 2016; Zhou, 2016; Hu and Y, 2018; Lu et al., 2019; Qiu and Wan, 2019; Wang and Bo, 2019; Liu, 2020).

**TABLE 1:** Basic characteristics of included trials and subjects.

References	Sample size (T/C)	Age (years)	Intervention	Control	Treatment duration	Follow-up duration	Outcome measures
Tong QL et al., 2023	126 (63/63)	T: 29.14 ± 5.23	CHM (*Fufang Xuanju* capsules)	Blank Control	CHM:1.26 g, tid, discontinue during menstruation, 4 weeks/course * 3 courses	12 months	①②③⑤
		C: 29.37 ± 6.02					
Wu L et al., 2021	106 (53/53)	T: 34.32 ± 2.35	CHM (*Cinnamon Twig and Poria* pills) + Leuprorelin	Leuprorelin	CHM:1.35 g, bid, discontinue during menstruation, 4 weeks/course * 6 courses	6 months	②③
		C: 34.96 ± 2.15			Leuprorelin: Once every 4 weeks * 6 courses		
Zhao XJ et al., 2020	116 (58/58)	T: 29.41 ± 5.27	CHM (*Yishen Shugan* decoction) +Triptorelin	Triptorelin	CHM:1 dose/d (100 mL, tid), discontinue during menstruation, 4 weeks/course * 6 courses	6 months	①②③④⑤
		C: 29.45 ± 5.21			Triptorelin: Once every 4 weeks * 6 courses		
Liu X 2020	66 (33/33)	T: 31.53 ± 6.09	CHM (*Huayu Xiaozheng* decoction) + Goserelin	Goserelin	CHM:1 dose/d (200 mL, bid), discontinue during menstruation, 14 days/course * 3 courses	7 months	①④⑤⑥
		C: 32.07 ± 4.81			Goserelin: Once every 28 days * 3 courses		
Qiu YF et al., 2019	68 (34/34)	T: 28.25 ± 6.12	CHM (*Wenshen Xiaozheng* decoction) + Triptorelin	Triptorelin	CHM:1 dose/d (150 mL, bid), start on Day 5 of Menstrual Cycle, 14 days/course * 3 courses	24 months	①⑤⑥
		C: 28.19 ± 6.04			Triptorelin: Once every 28 days * 6 courses		
Wang L et al., 2019	116 (58/58)	T: 32.15 ± 3.18	CHM (*Xiaojin* capsules) +Leuprorelin	Leuprorelin	CHM: 1.5 g, bid, 4 weeks/course * 6 courses	12 months	①②⑥
		C: 33.21 ± 3.68			Leuprorelin: Once every 4 weeks * 6 courses		
Song HP et al., 2019	160 (80/80)	T: 37.12 ± 2.59	CHM (*Kuntai* capsules) + Leuprorelin	Leuprorelin	CHM: 2 g, tid, 28 days/course * 3 courses	4 months	①③
		C: 36.25 ± 3.72			Leuprorelin: Once every 28days * 3 courses		
Lu YH et al., 2019	86 (43/43)	T: 29.17 ± 3.28	CHM (*Guizhi Fuling* capsules) + Gestrinone	Gestrinone	CHM: 0.93 g, tid, 4 weeks/course * 6 courses	6 months	②④⑥
		C: 28.76 ± 3.92			Gestrinone: 2.5 mg, qd, twice a week, 4 weeks/course * 6 courses		
Hu YY et al., 2018	90 (45/45)	T: 30.9 ± 5.54	CHM (*Dan’e Fukang* decocted extract) + Triptorelin	Triptorelin	CHM: 15 g, bid, start orally on Day 10 prior to menstruation, 14 days/course * 3 courses	6 months	①②③④⑥
		C: 30.20 ± 6.12			Triptorelin: Once every 28days * 3 courses		
Chen M et al., 2018	100 (50/50)	T: 31.6 ± 4.72	CHM (*Neiyi* decoction) + Triptorelin	Triptorelin	CHM: 1 dose/d (100 mL, bid), discontinue during menstruation, 4 weeks/course * 6 courses	9 months	②
		C: 30.90 ± 5.01			Triptorelin: Once every 4 weeks * 6 courses		
Zhou Q et al., 2016	106 (53/53)	T: 31.94 ± 2.80	CHM (*Fuzheng Xiaoyi* decoction) + Goserelin	Goserelin	CHM: 1 dose/d (100 mL, bid), 4 weeks/course * 3 courses	36 months	①②④⑥
		C: 32.64 ± 2.47			Goserelin: Once every 4 weeks * 6 courses		
Xing LM 2016	72 (36/36)	T: 29.64 ± 4.91	CHM (Empirical formula) + Mifepristone	Mifepristone	CHM: 1 dose/d (100 mL, bid), * 3 months	6 months	①③④⑥
		C: 30.56 ± 5.85			Mifepristone: 10 mg, qd, * 3 months		
Han B 2016	60 (30/30)	T: 30.35 ± 5.40	CHM (*Turtle Shell* decocted pills) + Leuprorelin	Leuprorelin	CHM: 3 g, tid, discontinue during menstruation, 28 days/course * 6 courses	6 months	①③④⑥
		C: 28.90 ± 5.49			Leuprorelin: Once every 28days * 6 courses		
Du X 2015	120 (60/60)	T: 31.72 ± 6.57	CHM (*Neiyi* decoction) + Triptorelin	Triptorelin	CHM:1 dose/d (200 mL, bid), start on Day 5 of Menstrual Cycle, 4 weeks/course * 6 courses	6 months	②④
		C: 33.76 ± 5.73			Triptorelin: Once every 4 weeks * 6 courses		
Chen LQ et al., 2015	48 (24/24)	T: 28.44 ± 2.37	CHM (*Xuefu Zhuyu* capsules) + Triptorelin	Triptorelin	CHM: 2.4 g, bid, 30 days/course * 3courses	3 months	①②③
		C: 27.49 ± 1.95			Triptorelin: Once every 28days * 5 courses		
Chen JJ 2015	72 (36/36)	T: 30.17 ± 4.08	CHM (*Bushen Huayu* decoction) + Mifepristone	Mifepristone	CHM: 1 dose/d (100 mL, bid), take for 1 week, then discontinue for 1 week, 4 weeks/course * 3 courses	6 months	①③④⑥
		C: 29.79 ± 4.15			Mifepristone: 10 mg, qd, 4 weeks/course * 3 courses		
Dou N 2015	70 (35/35)	T: 26.12 ± 4.21	CHM (*Danbie* capsules)	Gestrinone	CHM: 1.9 g, tid, discontinue during menstruation, 28 days/course * 6courses	12 months	①②④⑥
		C: 28.72 ± 6.14			Gestrinone: 2.5 mg, qd, twice a week, 4 weeks/course * 6 courses		
Li S 2014	60 (30/30)	T: 29.4 ± 5.30	CHM (Empirical formula)	Triptorelin	CHM: 1.5 dose/d (100 mL, bid), 10 days/course * 3 courses	12 months	①②⑤
		C: 28.9 ± 5.41			Triptorelin: Once every 28 days * 3 courses		
Zhou D et al., 2013	120 (60/60)	T: 34.1 ± 1.8	CHM (Empirical formula) + Mifepristone	Mifepristone	CHM: 1 dose/d (100 mL, qd), 4 weeks/course * 6 courses	6 months	①②⑥
		C: 34.5 ± 1.1			Mifepristone: 12.5 mg, qd, 4 weeks/course * 6 courses		
Zhang XN 2012	60 (30/30)	T: 29.70 ± 4.23	CHM (*Muda Tang* granules)	Gestrinone	CHM: 2 sachets, bid, discontinue during menstruation, 4 weeks/course * 3 courses	6 months	①③④⑥
		C: 29.87 ± 4.43			Gestrinone: 2.5 mg, qd, twice a week, 4 weeks/course * 3 courses		
Ding XQ et al., 2012	56 (27/29)	T: 35.84	CHM (Empirical formula)	Gestrinone	CHM: 1 dose/d (100 mL, tid), discontinue during menstruation, 4 weeks/course * 3 courses	36 months	②⑤⑥
		C: 34.54			Gestrinone: 2.5 mg, qd, twice a week, 4 weeks/course * 3 courses		
Ma L et al., 2011	60 (30/30)	T: 31.7 ± 4.79	CHM (*Xiaojie An* capsules) + Gestrinone	Gestrinone	CHM: 0.76 g, tid, 8 weeks/course * 3courses	36 months	1 ②
		C: 31.7 ± 4.79			Gestrinone: 2.5 mg, qd, twice a week, 8 weeks/course * 3 courses		

AbbreviationC: control; CHM: chinese herbal medicine; T: treatment; ‘bid’ (bis in die) means twice a day; ‘qd’ (quaque die) means once a day; ‘tid’ (ter in die) means three times a day. ①: Recurrence rate; ②: Total clinical efficacy rate; ③: Visual analog scale score; ④: Serum level of CA125; ⑤: Pregnancy rate; ⑥: Adverse events.

### 3.3 Assessment of methodological quality

**FIGURE 2:**
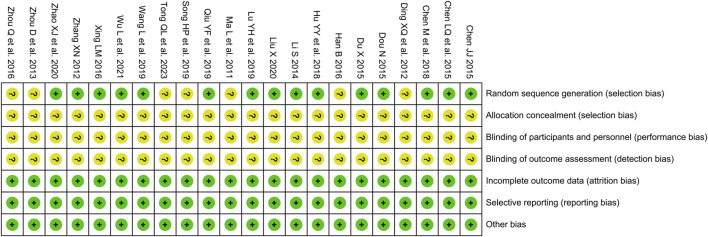
Risk of bias summary.

As shown in Figure 2, the methodological quality of the included studies was evaluated based on the criteria in the Cochrane handbook. Detailed information on the sequence generation of randomization was not reported in 7 trials (Ma, 2011; Ding and Shi, 2012; Zhou and Liu, 2013; Han, 2016; Zhou, 2016; Song et al., 2019; Tong et al., 2023). There were no statistically significant differences in baseline between the intervention and control groups across all enrolled studies. A specific method of allocation concealment was not described in this review. Detailed information regarding blinding of patients and investigators was unclear in all enrolled trials. Outcome data were obtained for nearly all randomized groups of subjects. All studies were free of bias from other sources. Although all studies were unclear on the blinding of outcome assessment, patients with OEC have objective evaluation indexes for recurrence, efficacy, pregnancy and serum CA-125 levels, and it was difficult to affect the outcome’s evaluation. No information mentioned that the results were analyzed in accordance with a published pre-specified analysis plan. Consistent outcome measures and data analysis methods were used for all included studies.

### 3.4 Description of single herb and CHM

Twenty-two CHM were used in this review, including five dosage formulations: decoction (11/22, 50.00%), capsule (7/22, 31.82%), pill (2/22, 9.10%), granule (1/22, 4.55%), and decocted extract (1/22, 4.55%). Decoction was the most commonly used formulation, accounting for the highest percentage. Table 1 illustrates the administration of CHM in each trial, and the specific components of CHM used in the included studies can be found in Supplementary Appendix S3.

The frequency of each Chinese herb in this review was also summarized manually. In total, 97 Chinese herbs were included, with a cumulative frequency of 236. Classified by CHM efficacies, they were divided into 15 categories. The top three categories were invigorate blood and dissolve stasis (81/236, 34.32%), supplement deficiency (61/236, 25.85%), and soothe the liver/rectify qi (32/236, 13.56%), refer to Table 2 for details. Additionally, specific components of CHM are listed in Table 3. The top 5 ranking CHM were *Prunus persica* (L.) Batsch [Rosaceae; Persicae Semen] (Taoren) (10/236, 4.24%), *Angelica sinensis* (Oliv.) Diels [Apiaceae; Angelicae Sinensis Radix] (Danggui) (9/236, 3.81%), *S. miltiorrhiza* Bunge [Lamiaceae; Salviae Miltiorrhizae Radix et Rhizoma] (Danshen) (9/236, 3.81%), *Paeonia lactiflora* Pall. [Paeoniaceae; Paeoniae Radix Rubra] (Chishao) (8/236, 3.39%), and *Corydalis yanhusuo* (Y.H.Chou & Chun C.Hsu) W.T.Wang ex Z.Y.Su and C.Y.Wu [Papaveraceae; Corydalis Rhizoma] (Yanhusuo) (7/236, 2.97%). The top three CHM natures were warm (92/236, 38.98%), cold (79/236, 33.47%), and neutral (63/236, 26.69%), while the top three flavors were bitter (132/389, 33.93%), sweet (111/389, 28.53%), and acrid (103/389, 26.48%). In terms of channel entries, the top four were the foot *jueyin* liver channel (181/598, 30.27%), the hand *shaoyin* heart channel (93/598, 15.55%), the foot *taiyin* spleen channel (88/598, 14.72%), and the foot *shaoyin* kidney channel (80/598, 13.38%). Further details can be found in Table 4.

**TABLE 2:** Categorization of Chinese herbal medicine efficacies in the included studies.

Cat	Qty	Freq	Pct.(%)	Cat	Qty	Freq	Pct.(%)
Invigorate blood and dissolve stasis	21	81	34.32	Resolve toxin and dissipate binds	5	5	2.12
Supplement deficiency	23	61	25.85	Calm the liver and extinguish wind	2	5	2.12
Soothe the liver/Rectify qi	13	32	13.56	Promote digestion and resolve stagnation	2	4	1.69
Heat-clearing	11	14	5.93	Warm the interior	2	2	0.85
Dissolve phlegm	5	9	3.81	Promote astriction	2	2	0.85
Promote urination and percolate dampness	3	7	2.97	Dispel wind and overcome dampness	2	2	0.85
Release the exterior and dissipate cold	1	6	2.54	Open the orifices	1	1	0.42
Stanch bleeding	4	5	2.12				

**Abbreviation:** Cat.: category; CHM: chinese herbal medicine; Freq: Frequency; Pct.: percentage; Qty.: quantity.

**TABLE 3:** Frequency of Chinese herbal medicine Utilization in the Included Studies.

Full name	Chinese name	Freq	Pct. (%)	Full name	Chinese name	Freq	Pct. (%)	Full name	Chinese name	Freq	Pct. (%)
*Prunus persica* (L.) Batsch [Rosaceae; Persicae Semen]	Taoren	10	4.24	*Trionyx sinensis* Wiegmann [Trionychidae; Trionycis Carapax]	Biejia	2	0.85	*Liquidambar formosana* Hance [Altingiaceae; Liquidambaris Resina]	Fengxiangzhi	1	0.42
*Angelica sinensis* (Oliv.) Diels [Apiaceae; Angelicae Sinensis Radix]	Danggui	9	3.81	*Equus asinus* L. [Equidae; Asini Corii Colla]	Ejiao	2	0.85	*Boswellia sacra* Flück. [Burseraceae; Olibanum]	Ruxiang	1	0.42
*Salvia miltiorrhiza* Bunge [Lamiaceae; Salviae Miltiorrhizae Radix et Rhizoma]	Danshen	9	3.81	*Lycium barbarum* L. [Solanaceae; Lycii Fructus]	Gouqi	2	0.85	*Campsis grandiflora* (Thunb.) K.Schum. [Bignoniaceae; Campsis Flos]	Lingxiaohua	1	0.42
*Paeonia lactiflora* Pall. [Paeoniaceae; Paeoniae Radix Rubra]	Chishao	8	3.39	*Cervi Cornu Degelatinatum*	Lujiaoshuang	2	0.85	*Achyranthes bidentata* Blume [Amaranthaceae; Achyranthis Bidentatae Radix]	Niuxi	1	0.42
*Corydalis yanhusuo* (Y.H.Chou & Chun C.Hsu) W.T.Wang ex Z.Y.Su and C.Y.Wu [Papaveraceae; Corydalis Rhizoma]	Yanhusuo	7	2.97	*Panax notoginseng* (Burkill) F.H.Chen [Araliaceae; Notoginseng Radix et Rhizoma]	Sanqi	2	0.85	*Moschus berezovskii* Flerov [Moschidae; Moschus]	Shexiang	1	0.42
*Neolitsea cassia* (L.) Kosterm. [Lauraceae; Cinnamomi Ramulus]	Guizhi	6	2.54	*Carthamus tinctorius* L. [Asteraceae; Carthami Flos]	Honghua	2	0.85	*Citrus reticulata* Blanco [Rutaceae; Citri Reticulatae Pericarpium]	Chenpi	1	0.42
*Sparganium stoloniferum* (Buch.-Ham. ex Graebn.) Buch.-Ham. ex Juz. [Typhaceae; Sparganii Rhizoma]	Sanleng	6	2.54	*Calamus draco* Willd. [Arecaceae; Draconis Sanguis]	Xuejie	2	0.85	*Magnolia officinalis* Rehder &E.H.Wilson [Magnoliaceae; Magnoliae Officinalis Cortex]	Houpo	1	0.42
*Curcuma longa* L. [Zingiberaceae; Curcumae Rhizoma]	Ezhu	6	2.54	*Lindera aggregata* (Sims) Kosterm. [Lauraceae; Linderae Radix]	Wuyao	2	0.85	*Citrus reticulata* Blanco [Rutaceae; Citri Reticulatae Pericarpium Viride]	Qingpi	1	0.42
*Paeonia × suffruticosa* Andrews [Paeoniaceae; Moutan Cortex]	Mudanpi	6	2.54	*Citrus × aurantium f. aurantium* [Rutaceae; Aurantii Fructus]	Zhiqiao	2	0.85	*Citrus reticulata* Blanco [Rutaceae; Citri Reticulatae Semen]	Juhe	1	0.42
*Cyperus rotundus* L. [Cyperaceae; Cyperi Rhizoma]	Xiangfu	6	2.54	*Smilax glabra* Roxb. [Smilacaceae; Smilacis Glabrae Rhizoma]	Tufuling	2	0.85	*Melia azedarach* L. [Meliaceae; Toosendan Fructus]	Chuanlianzi	1	0.42
*Cuscuta chinensis* Lam. [Convolvulaceae; Cuscutae Semen]	Tusizi	5	2.12	*Scutellaria baicalensis* Georgi [Lamiaceae; Scutellariae Radix]	Huangqin	2	0.85	*Dolomiaea costus* (Falc.) Kasana and A.K.Pandey [Asteraceae; Aucklandiae Radix]	Muxiang	1	0.42
*Glycyrrhiza glabra* L. [Fabaceae; Glycyrrhizae Radix et Rhizoma]	Gancao	5	2.12	*Rheum officinale* Baill. [Polygonaceae; Rhei Radix et Rhizoma]	Dahuang	2	0.85	*Platycodon grandiflorus* (Jacq.) A.DC. [Campanulaceae; Platycodonis Radix]	Jiegeng	1	0.42
*Conioselinum anthriscoides ‘Chuanxiong’* [Apiaceae; Chuanxiong Rhizoma]	Chuanxiong	5	2.12	*Prunus mume* (Siebold) Siebold & Zucc. [Rosaceae; Mume Fructus]	Wumei	2	0.85	*Pyrrosia lingua* (Thunb.) Farw. [Polypodiaceae; Pyrrosiae Folium]	Shiwei	1	0.42
*Bupleurum chinense* DC. [Apiaceae; Bupleuri Radix]	Chaihu	5	2.12	*Gallus gallus domesticus* Brisson [Phasianidae; Galli Gigerii Endothelium Corneum]	Jineijin	2	0.85	*Dianthus chinensis* L. [Caryophyllaceae; Dianthi Herba]	Qumai	1	0.42
*Carapichea ipecacuanha* (Brot.) L.Andersson [Rubiaceae; Poria]	Fuling	5	2.12	*Crataegus monogyna* Jacq. [Rosaceae; Crataegi Fructus]	Shanzha	2	0.85	Fossilia Ossis Mastodi	Longgu	1	0.42
*Dipsacus asper* Wall. Ex DC. [Caprifoliaceae; Dipsaci Radix]	Xuduan	4	1.69	*Typha angustifolia* L. [Typhaceae; Typhae Pollen]	Puhuang	2	0.85	*Pheretima aspergillum* (E.Perrier)[Pheretimidae; Pheretima]	Dilong	1	0.42
*Paeonia lactiflora* Pall. [Paeoniaceae; Paeoniae Radix Alba]	Baishao	4	1.69	*Cibotium barometz* (L.) J.Sm. [Cibotiaceae; Cibotii Rhizoma]	Gouji	1	0.42	*Abutilon indicum* (L.) Sweet [Malvaceae]	Mopancao	1	0.42
*Astragalus mongholicus* Bunge [Fabaceae; Astragali Radix]	Huangqi	4	1.69	*Rubus chingii* Hu [Rosaceae; Rubi Fructus]	Fupenzi	1	0.42	*Coptis chinensis* Franch. [Ranunculaceae; Coptidis Rhizoma]	Huanglian	1	0.42
*Eupolyphaga sinensis* Walker [Corydiidae; Eupolyphaga Steleophaga]	Tubiechong	4	1.69	*Cornus officinalis* Siebold & Zucc. [Cornaceae; Corni Fructus]	Shanzhuyu	1	0.42	*Iris domestica* (L.) Goldblatt & Mabb. [Iridaceae; Belamcandae Rhizoma]	Shegan	1	0.42
*Trogopterori Faeces*	Wulingzhi	4	1.69	*Ganoderma lucidum* (Curtis) P.Karst.[Polyporaceae; Ganoderma]	Lingzhi	1	0.42	*Lobelia chinensis* Lour. [Campanulaceae; Lobeliae Chinensis Herba]	Banbianlian	1	0.42
*Gynochthodes officinalis* (F.C.How) Razafim. and B.Bremer [Rubiaceae; Morindae Officinalis Radix]	Bajitian	3	1.27	*Rhodiola crenulata* (Hook.f. and Thomson) H.Ohba [Crassulaceae; Rhodiolae Crenulatae Radix et Rhizoma]	Hongjingtian	1	0.42	*Scutellaria barbata* D.Don [Lamiaceae; Scutellariae Barbatae Herba]	Banzhilian	1	0.42
*Rehmannia glutinosa* (Gaertn.) DC. [Orobanchaceae; Rehmanniae Radix Praeparata]	Shudi	3	1.27	*Atractylodes macrocephala* Koidz. [Asteraceae; Atractylodis Macrocephalae Rhizoma]	Baizhu	1	0.42	*Berberis bealei* Fortune [Berberidaceae; Mahoniae Caulis]	Gonglaomu	1	0.42
*Eucommia ulmoides* Oliv. [Eucommiaceae; Eucommiae Cortex]	Duzhong	3	1.27	*Cnidium monnieri* (L.) Cusson [Apiaceae; Cnidii Fructus]	Shechuangzi	1	0.42	*Melicope pteleifolia* (Champ. ex Benth.) T.G.Hartley [Rutaceae]	Sanchaku	1	0.42
*Epimedium sagittatum* (Siebold & Zucc.) Maxim. [Berberidaceae; Epimedii Folium]	Yinyanghuo	3	1.27	*Codonopsis pilosula* (Franch.) Nannf. [Campanulaceae; Codonopsis Radix]	Dangshen	1	0.42	*Forsythia suspensa* (Thunb.) Vahl [Oleaceae; Forsythiae Fructus]	Lianqiao	1	0.42
*Sargassum pallidum* (Turn.) C.Ag. [Sargassaceae; Sargassum]	Haizao	3	1.27	*Bolbostemma paniculatum* (Maxim.) Franquet [Cucurbitaceae; Bolbostematis Rhizoma]	Tubeimu	1	0.42	*Liquidambar formosana* Hance [Altingiaceae; Liquidambaris Fructus]	Lulutong	1	0.42
*Fritillaria thunbergii* Miq. [Liliaceae; Fritillariae Thunbergii Bulbus]	Zhebeimu	3	1.27	*Momordica cochinchinensis* (Lour.) Spreng. [Cucurbitaceae; Momordicae Semen]	Mubiezi	1	0.42	*Aconitum kusnezoffii* Rchb.[Ranunculaceae; Aconiti Kusnezoffii Radix Cocta]	Zhicaowu	1	0.42
*Curcuma longa* L. [Zingiberaceae; Curcumae Radix]	Yujin	3	1.27	*Polistes olivaceous* (DeGeer) [Eumenidae; Vespae Nidus]	Fengfang	1	0.42	*Zingiber officinale* Roscoe [Zingiberaceae; Zingiberis Rhizoma]	Ganjiang	1	0.42
*Leonurus japonicus* Houtt. [Lamiaceae; Leonuri Herba]	Yimucao	3	1.27	*Catharsius molossus* Linnaeus [Scarabaeidae]	Qianglang	1	0.42	*Foeniculum vulgare* Mill. [Apiaceae; Foeniculi Fructus]	Xiaohuixiang	1	0.42
*Spatholobus suberectus* Dunn [Fabaceae; Spatholobi Caulis]	Jixueteng	3	1.27	Nitrum	Xiaoshi	1	0.42	Atramentum	Xiangmo	1	0.42
*Commiphora myrrha* (T.Nees) Engl. [Burseraceae; Myrrha]	Moyao	3	1.27	*Pinellia ternata* (Thunb.) Makino [Araceae; Pinelliae Rhizoma]	Banxia	1	0.42	*Cirsium japonicum* DC. [Asteraceae; Cirsii Japonici Herba]	Daji	1	0.42
*Litchi chinensis* Sonn. [Sapindaceae; Litchi Semen]	Lizhihe	3	1.27	*Descurainia sophia* (L.) Webb ex Prantl [Brassicaceae; Descurainiae Semen]	Tinglizi	1	0.42	*Cirsium arvense* var. *arvense* [Asteraceae; Cirsii Herba]	Xiaoji	1	0.42
*Ostrea gigas* Thunberg [Ostreidae; Ostreae Concha]	Muli	3	1.27	*Gleditsia sinensis* Lam. [Fabaceae; Gleditsiae Spina]	Zaojiaoci	1	0.42				
*Taxillus chinensis* (DC.) Danser [Loranthaceae; Taxilli Herba]	Sangjisheng	2	0.85	*Euonymus alatus* (Thunb.) Siebold [Celastraceae]	Guijianyu	1	0.42				

**Abbreviation:** Freq: Frequency; Pct.: percentage.

**TABLE 4:** Analysis of Chinese herbal medicine natures, flavors, and channel entries in the included studies.

Medicinal nature	Freq	Pct.(%)	Medicinal flavor	Freq	Pct.(%)	Channel entry	Freq	Pct.(%)
Warm	92	38.98	Bitter	132	33.93	the foot *jueyin* liver channel	181	30.27
Cold	79	33.47	Sweet	111	28.53	the hand *shaoyin* heart channel	93	15.55
Neutral	63	26.69	Acrid	103	26.48	the foot *taiyin* spleen channel	88	14.72
Hot	2	0.85	Salty	23	5.91	the foot *shaoyin* kidney channel	80	13.38
			Sour	12	3.08	the hand *taiyin* lung channel	49	8.19
			Astringent	8	2.06	the foot *yangming* stomach channel	36	6.02
						the hand *yangming* large intestine channel	22	3.68
						the foot *taiyang* bladder channel	19	3.18
						the foot *shaoyang* gallbladder channel	17	2.84
						the hand *taiyang* small intestine channel	6	1.00
						the hand *jueyin* pericardium channel	4	0.67
						the hand *shaoyang* san jiao channel	3	0.50

Abbreviation Freq: Frequency; Pct.: percentage.

### 3.5 Efficacy assessment

#### 3.5.1 Recurrence rate

Current guidelines for diagnosing and treating endometriosis suggest that patients with postoperative recurrence of OEC may experience similar or exacerbated clinical symptoms (Gynecology and Obsterics, 2019; Association, 2021). Bimanual gynecological examinations have identified cystic masses in the bilateral adnexal regions and palpable nodules in the posterior vaginal fornix, which are often tender. Additionally, ultrasounds have detected cysts in the adnexal area (Gynecology and Obstetrics, 2019; Association, 2021; Becker et al., 2022). Of the 22 studies reviewed, 17 addressed OEC recurrence rates. Eight of these studies defined recurrence as the reappearance of ovarian endometriotic lesions via ultrasound examination (Zhang, 2012; Han, 2016; Zhou, 2016; Hu and Y, 2018; Wang and Bo, 2019; Liu, 2020; Zhao and Xiao, 2020; Tong et al., 2023). Seven studies defined OEC recurrence as the reappearance of both endometriotic lesions and clinical symptoms (Zhou and Liu, 2013; Li, 2014; Chen, 2015; Chen et al., 2015; Dou, 2015; Xing, 2016; Qiu and Wan, 2019). Meanwhile, two studies characterized OEC recurrence as either the recurrence of endometriotic lesions or clinical symptoms (Ma, 2011; Song et al., 2019). Recurrence rate was quantified as the ratio of recurrent cases to the total patient count (Ma, 2011; Zhang, 2012; Zhou and Liu, 2013; Li, 2014; Chen, 2015; Chen et al., 2015; Dou, 2015; Han, 2016; Xing, 2016; Zhou, 2016; Hu and Y, 2018; Qiu and Wan, 2019; Song et al., 2019; Wang and Bo, 2019; Liu, 2020; Zhao and Xiao, 2020; Tong et al., 2023).

##### 3.5.1.1 CHM alone vs blank control

**FIGURE 3:**
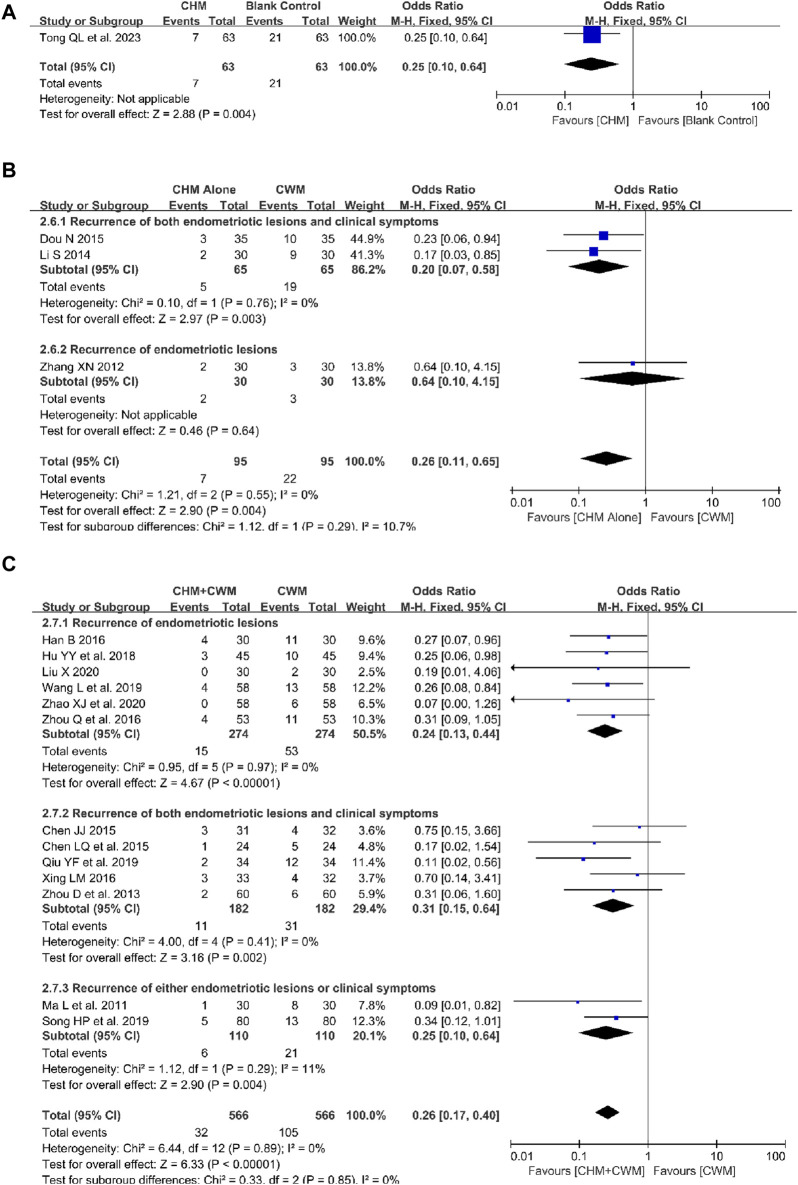
Forest plot illustrating the effect on recurrence rate of different intervention modes of CHM: **(A)** CHM Alone vs Blank Control, **(B)** CHM Alone vs CWM, **(C)** CHM + CWM vs CWM.

In a comparative analysis of CHM *versus* a blank control, one study assessed the recurrence rates of OEC in groups of 63 patients each (Tong et al., 2023). Recurrence was identified by the reappearance of endometriotic lesions. The result demonstrated that CHM was superior to blank control in decreasing the recurrence rate (1 trial, n = 126; OR = 0.25; 95% CI: 0.10–0.64; *p* = 0.004; Figure 3A).

##### 3.5.1.2 CHM alone vs CWM

The comparative impact of CHM *versus* CWM on recurrence rates was assessed across three studies (Zhang, 2012; Li, 2014; Dou, 2015). As shown in Figures 2A,B fixed-effect model was used due to no obvious heterogeneity observed. Meta-analysis showed that the overall recurrence rate was lower with CHM alone than with CWM (3 trials, n = 190; OR = 0.26; 95% CI: 0.11–0.65; *I* ^*2*^ = 0%*, p* = 0.004; Figure 3B). Furthermore, in two of these studies, recurrence was specifically defined as the reappearance of both endometriotic lesions and clinical symptoms (Li, 2014; Dou, 2015), showing the CHM group to have a significantly lower postoperative recurrence rate than the CWM group (2 trials, n = 130; OR = 0.20; 95% CI: 0.07–0.58; *I* ^*2*^ = 0%*, p* = 0.003; Figure 3B). Conversely, one study identified recurrence solely based on the return of endometriotic lesions (Zhang, 2012), with the analysis revealing no significant difference in efficacy between CHM and CWM in reducing recurrence rates (1 trial, n = 60; OR = 0.64; 95% CI: 0.10–4.15; *p* = 0.64; Figure 3B).

##### 3.5.1.3 CHM + CWM vs CWM

Thirteen studies evaluated the effectiveness of combining CHM with CWM in reducing the recurrence rate of OEC (Ma, 2011; Zhou and Liu, 2013; Chen, 2015; Chen et al., 2015; Han, 2016; Xing, 2016; Zhou, 2016; Hu and Y, 2018; Qiu and Wan, 2019; Song et al., 2019; Wang and Bo, 2019; Liu, 2020; Zhao and Xiao, 2020). These trials included a total of 1,132 patients, evenly split between the combination treatment and CWM-only groups. The absence of heterogeneity across the studies supported a uniform analysis. The results indicate that the combination of CHM with CWM significantly reduced the overall postoperative recurrence rate of OEC (13 trials, n = 1,132; OR = 0.26; 95% CI: 0.17–0.40; *I* ^*2*^ = 0%*, p* < 0.00001; Figure 3C). The analysis further categorized the studies into three groups based on their recurrence definitions, demonstrating the combination therapy’s significant advantage in reducing recurrence rates. The benefits were evident in (a) reducing the recurrence of endometriotic lesions alone (Han, 2016; Zhou, 2016; Hu and Y, 2018; Wang and Bo, 2019; Liu, 2020; Zhao and Xiao, 2020) (6 trials, n = 548; OR = 0.24; 95% CI: 0.13–0.44; *I* ^*2*^ = 0%*, p* < 0.00001; Figure 3C), (b) reducing the recurrence of both endometriotic lesions and clinical symptoms (Zhou and Liu, 2013; Chen, 2015; Chen et al., 2015; Xing, 2016; Qiu and Wan, 2019) (5 trials, n = 364; OR = 0.31; 95% CI: 0.15–0.64; *I* ^*2*^ = 0%*, p* = 0.002; Figure 3C), and (c) reducing the recurrence of either endometriotic lesions or clinical symptoms (Ma, 2011; Song et al., 2019) (2 trials, n = 220; OR = 0.25; 95% CI: 0.10–0.64; *I* ^*2*^ = 11%*, p* = 0.004; Figure 3C).

#### 3.5.2 Total clinical efficacy rate

The total clinical efficacy rate was evaluated in sixteen studies (Ma, 2011; Ding and Shi, 2012; Zhou and Liu, 2013; Li, 2014; Chen et al., 2015; Dou, 2015; Du, 2015; Zhou, 2016; Chen, 2018; Hu and Y, 2018; Lu et al., 2019; Song et al., 2019; Wang and Bo, 2019; Zhao and Xiao, 2020; Wu and Deng, 2021; Tong et al., 2023). Eleven of these studies (Ma, 2011; Zhou and Liu, 2013; Chen et al., 2015; Dou, 2015; Zhou, 2016; Chen, 2018; Hu and Y, 2018; Song et al., 2019; Wang and Bo, 2019; Zhao and Xiao, 2020; Wu and Deng, 2021) defined efficacy as follows: (a) significantly effective: complete lesion resolution and symptom relief; (b) effective: lesion size reduction and symptom alleviation; (c) ineffective: no symptom improvement or exacerbation, along with OEC recurrence. Furthermore, five studies (Ding and Shi, 2012; Li, 2014; Du, 2015; Lu et al., 2019; Tong et al., 2023) classified efficacy into four categories: (a) cured: total lesion disappearance and symptom resolution; (b) significantly effective: symptom resolution and lesion size reduction; (c) effective: symptom alleviation without notable lesion size change; (d) ineffective: no improvement or symptom exacerbation, alongside OEC recurrence. Despite slight variations in the efficacy evaluation criteria across these studies, the definition of “ineffective” remains consistent. Furthermore, all studies employ a uniform method to calculate the total clinical efficacy rate, defined as the proportion of effective cases (calculated by subtracting the number of ineffective cases from the total patient count) to the overall patient population, facilitating meta-analysis.

##### 3.5.2.1 CHM alone vs blank control

**FIGURE 4:**
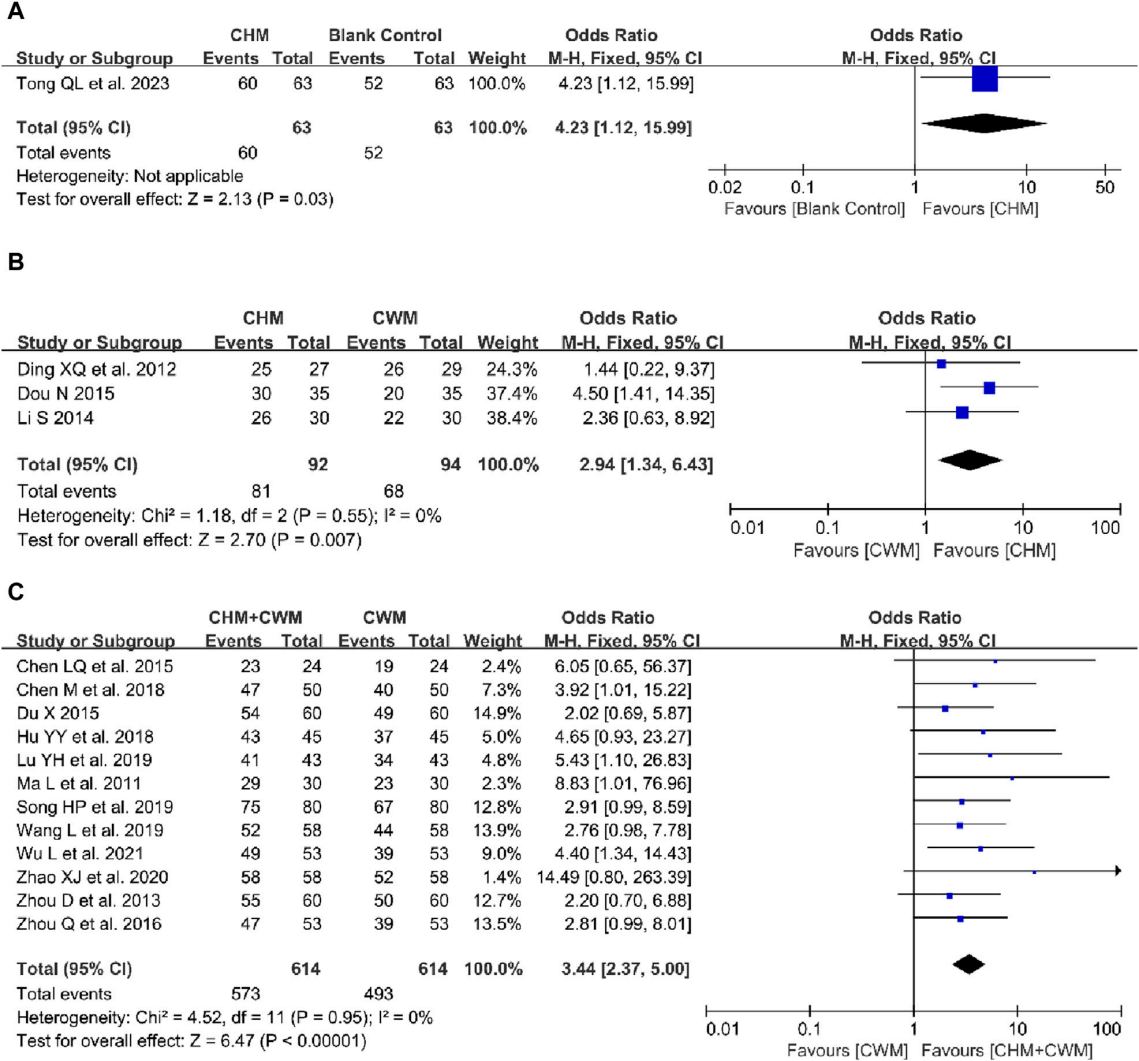
Forest plot illustrating the effect on total clinical efficacy rate of different intervention modes of CHM: **(A)** CHM Alone vs Blank Control, **(B)** CHM Alone vs CWM, **(C)** CHM + CWM vs CWM.

A single study evaluated the impact of CHM alone compared to a blank control on the overall clinical efficacy rate (Tong et al., 2023). The result indicated that CHM outperformed the blank control in improving clinical efficacy (1 trial, n = 126; OR = 4.23; 95% CI: 1.12–15.99; *p* = 0.03; Figure 4A).

##### 3.5.2.2 CHM alone vs CWM

The comparative efficacy of CHM alone and CWM on the overall clinical efficacy rate was assessed in three studies (Ding and SHI, 2012; Li, 2014; Dou, 2015). With no notable heterogeneity detected between the studies, a fixed-effects model was applied. Meta-analysis revealed that CHM achieved a higher clinical efficacy rate than CWM (3 trials, n = 186; OR = 2.94; 95% CI: 1.34–6.43; *I* ^*2*^ = 0%, *p* = 0.007; Figure 4B).

##### 3.5.2.3 CHM + CWM vs CWM

A meta-analysis of twelve studies (Ma, 2011; Zhou and Liu, 2013; Chen et al., 2015; Du, 2015; Zhou, 2016; Chen, 2018; Hu and Y, 2018; Lu et al., 2019; Wang and Bo, 2019; Zhao and Xiao, 2020; Wu and Deng, 2021) evaluated the combined effect of CHM and CWM *versus* CWM alone on the total clinical efficacy rate. The analysis included 614 patients in each of the combination and CWM-only groups, with no significant heterogeneity detected across the studies. The findings demonstrated that the combination of CHM and CWM significantly enhanced the overall clinical efficacy rate compared to CWM alone (12 trials, n = 1,228; OR = 3.44; 95% CI: 2.37–5.00; *I* ^*2*^ = 0%, *p* < 0.00001; Figure 4C).

#### 3.5.3 VAS score

##### 3.5.3.1 CHM alone vs blank control

**FIGURE 5:**
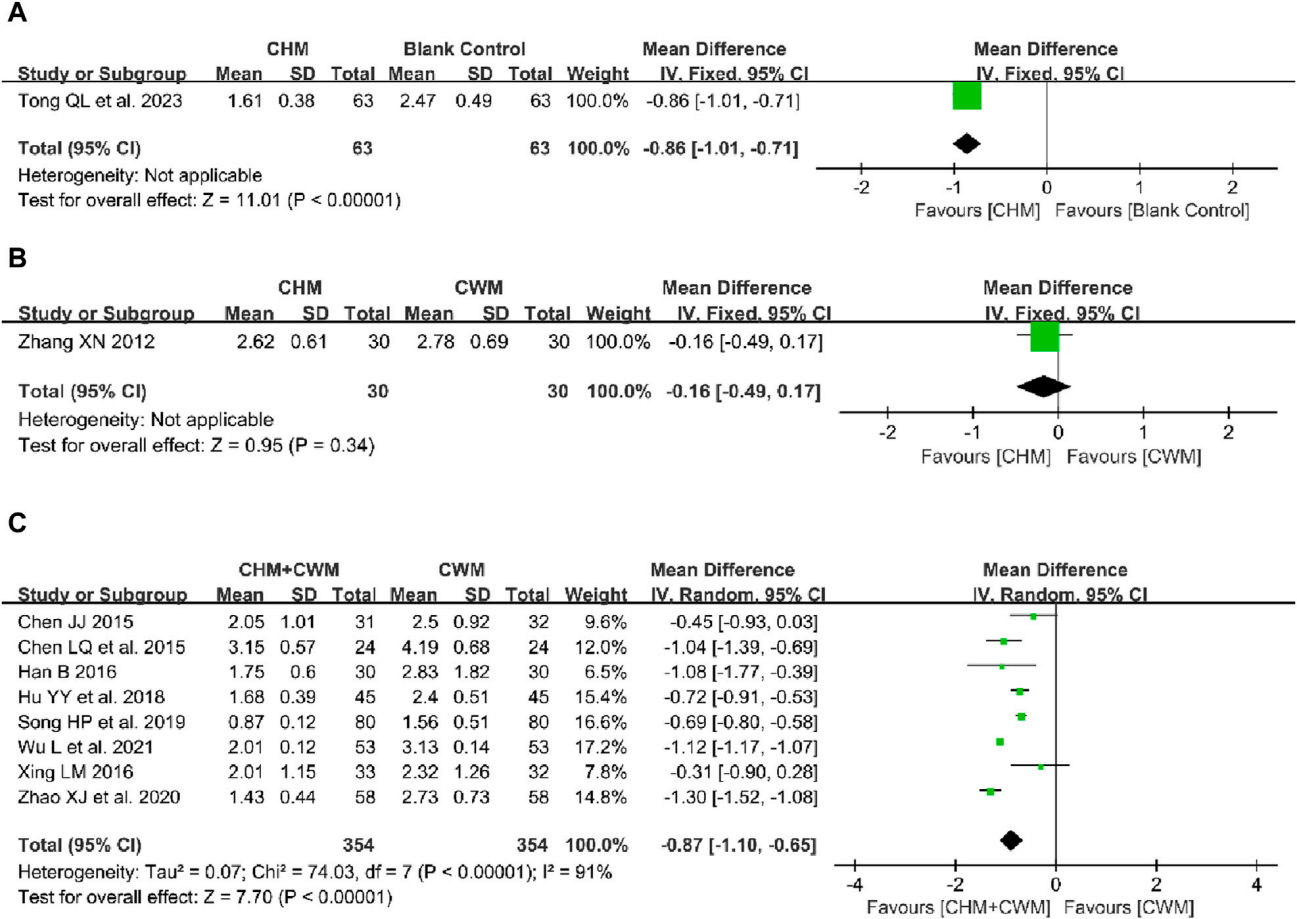
Forest plot illustrating the effect on VAS score of different intervention modes of CHM: **(A)** CHM Alone vs Blank Control, **(B)** CHM Alone vs CWM, **(C)** CHM + CWM vs CWM.

One study reported the effect of CHM alone *versus* a blank control on VAS scores, involving 126 patients (Tong et al., 2023). The result indicated that CHM could ameliorate postoperative pain symptoms in patients with OEC (1 trial, n = 126; MD = −0.86; 95% CI: -1.01 to −0.71; *p* < 0.00001; Figure 5A).

##### 3.5.3.2 CHM alone vs CWM

The effect of CHM alone *versus* CWM on VAS score was evaluated in 1 trial involving 60 patients (Zhang, 2012). The result showed that there was no significant difference between CHM and CWM on VAS score (1 trial, n = 60; MD = −0.16; 95% CI: -0.49 to 0.17; *p* = 0.34; Figure 5B).

##### 3.5.3.3 CHM + CWM vs CWM

Eight studies compared the variation in VAS scores between intervention and control groups (Chen, 2015; Chen et al., 2015; Han, 2016; Xing, 2016; Hu and Y, 2018; Song et al., 2019; Zhao and Xiao, 2020; Wu and Deng, 2021). There were 354 patients in the combination group and 354 patients in the CWM group. As depicted in Figure 5C, meta-analysis using a random-model suggested that CHM combined with CWM remarkably reduced the VAS score compared to CWM alone (8 trials, n = 708; MD = −0.87; 95% CI: -1.10 to −0.65; *I* ^*2*^ = 91%, *p* < 0.00001; Figure 5C).

#### 3.5.4 Serum level of CA125

##### 3.5.4.1 CHM alone vs CWM

**FIGURE 6:**
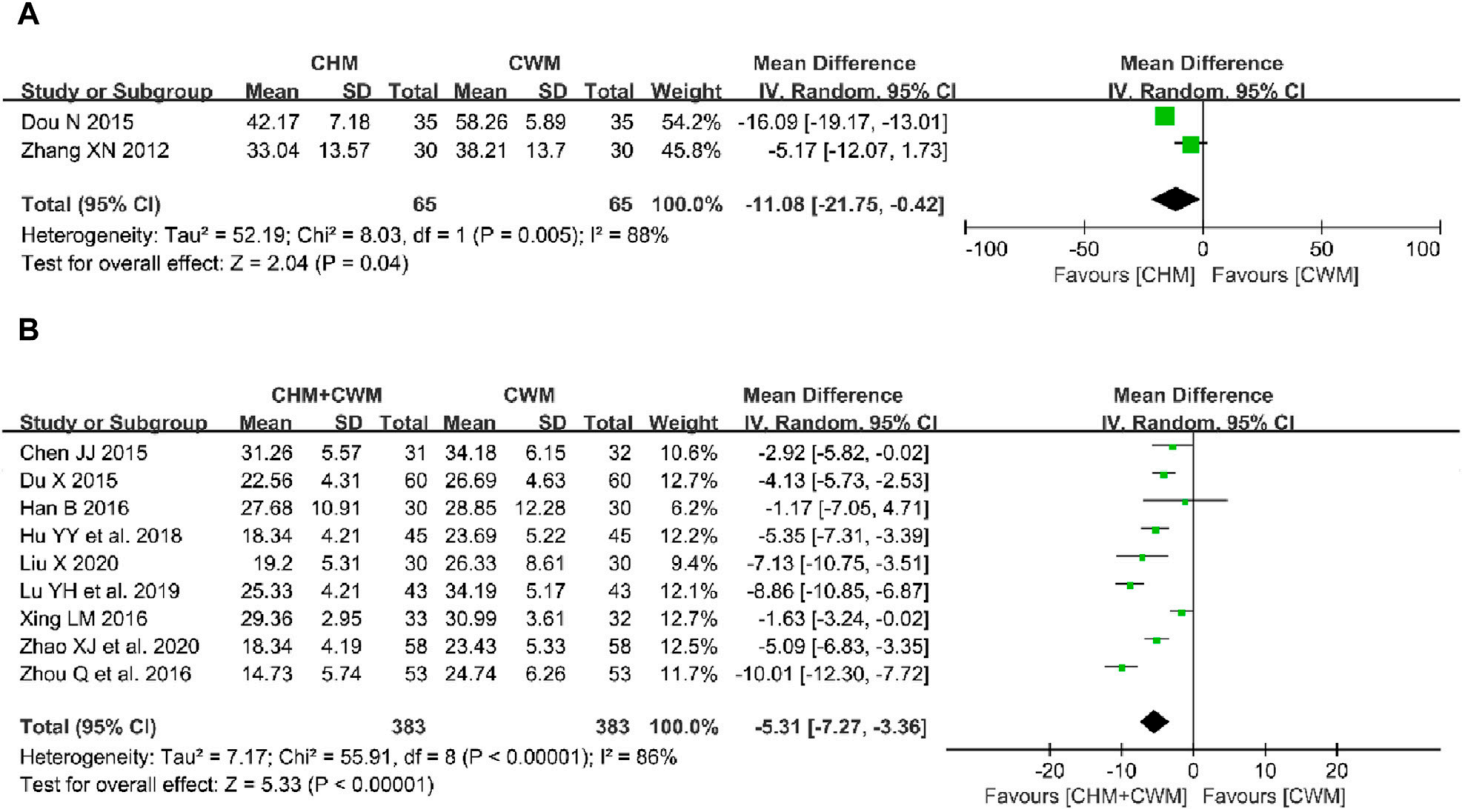
Forest plot illustrating the effect on serum level of CA125 of different intervention modes of CHM: **(A)** CHM Alone vs CWM, **(B)** CHM + CWM vs CWM.

The efficacy of CHM alone compared to CWM on serum level of CA125 was assessed in two studies involving 130 patients (Zhang, 2012; Dou, 2015). Meta-analysis revealed that the serum CA125 level was lower with CHM treatment than with CWM (2 trials, n = 130; MD = −11.08; 95% CI: -21.75 to −0.42; *I* ^*2*^ = 88%, *p* = 0.04; Figure 6A).

##### 3.5.4.2 CHM + CWM vs CWM

Nine studies evaluated the effect of CHM combined with CWM on serum level of CA125 (Chen, 2015; Du, 2015; Han, 2016; Xing, 2016; Zhou, 2016; Hu and Y, 2018; Lu et al., 2019; Liu, 2020; Zhao and Xiao, 2020). Each group, the combined treatment and CWM alone, included 383 patients. A meta-analysis employing a random-effects model demonstrated a significant reduction in serum CA125 levels with the CHM and CWM combination (9 trials, n = 766; MD = −5.31; 95% CI: -7.27 to −3.36; *I* ^*2*^ = 86%, *p* < 0.00001; Figure 6B).

#### 3.5.5 Pregnancy rate

##### 3.5.5.1 CHM alone vs blank control

**FIGURE 7:**
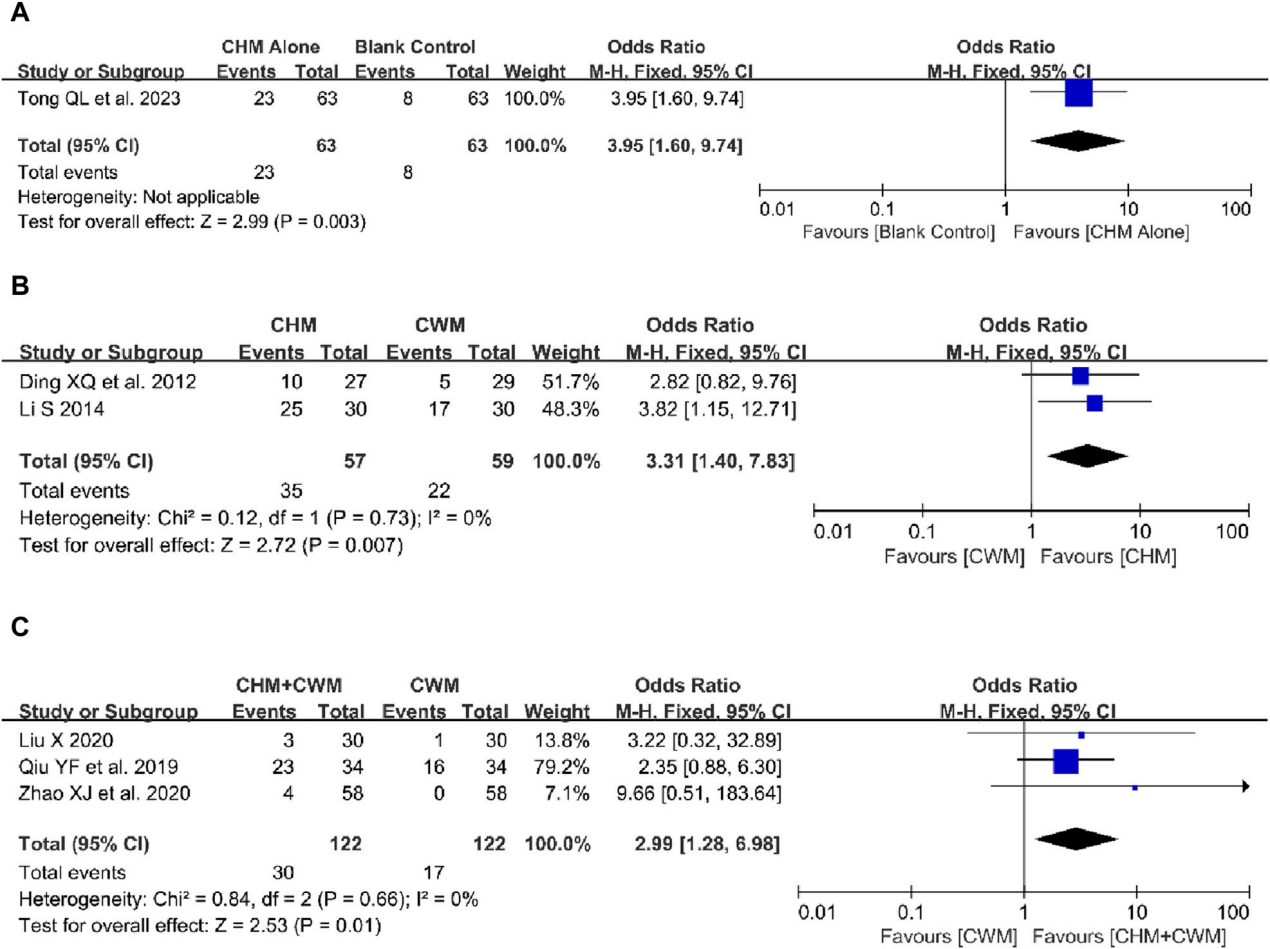
Forest plot illustrating the effect on pregnancy rate of different intervention modes of CHM: **(A)** CHM Alone vs Blank Control, **(B)** CHM Alone vs CWM, **(C)** CHM + CWM vs CWM.

One study reported the effect of CHM alone compared to a blank control on pregnancy rate, involving 126 patients (Tong et al., 2023). The result showed that CHM could increase the pregnancy rate in postoperative patients with OEC (1 trial, n = 126; OR = 3.95; 95% CI: 1.60–9.74; *p* = 0.003; Figure 7A).

##### 3.5.5.2 CHM alone vs CWM

As shown in Figure 7B, we applied a fixed-effect model because no obvious heterogeneity was observed. The result suggested that the postoperative pregnancy rate with CHM was higher than with CWM (Ding and Shi, 2012; Li, 2014) (2 trials, n = 116; OR = 3.31; 95% CI: 1.40–7.83; *I* ^*2*^ = 0%, *p* = 0.007; Figure 7B).

##### 3.5.5.3 CHM + CWM vs CWM

Three studies evaluated the impact of combining CHM with CWM on the pregnancy rate (Qiu and Wan, 2019; Liu, 2020; Zhao and Xiao, 2020). The analysis included 122 patients in each of the combination and CWM-only groups, with no significant heterogeneity across the studies. The combined treatment of CHM and CWM was found to significantly enhance the pregnancy rate (3 trials, n = 244; OR = 2.99; 95% CI: 1.28–6.98; *I* ^*2*^ = 0%, *p* = 0.01; Figure 7C).

#### 3.5.6 Adverse events

In this review, adverse events were reported in 14 of 22 studies (63.64%) (Ding and Shi, 2012; Zhang, 2012; Zhou and Liu, 2013; Chen, 2015; Dou, 2015; Du, 2015; Han, 2016; Xing, 2016; Zhou, 2016; Hu and Y, 2018; Lu et al., 2019; Qiu and Wan, 2019; Wang and Bo, 2019; Liu, 2020). Within these, two trials (Lu et al., 2019; Wang and Bo, 2019) found no adverse effects in either the CHM or CWM groups. The remaining 12 studies documented various adverse effects, including perimenopausal symptoms (such as amenorrhea, hot flashes, irritability, decreased libido, insomnia, and irregular vaginal bleeding), androgenic response (acne, weight gain, breast reduction, edema), gastrointestinal discomfort (nausea, vomiting), physical pain (headache, breast pain, limb joint pain, muscle pain), allergic reactions (itching of the skin, urticaria, rash), and hepatic function impairment.

##### 3.5.6.1 CHM alone vs CWM

**FIGURE 8:**
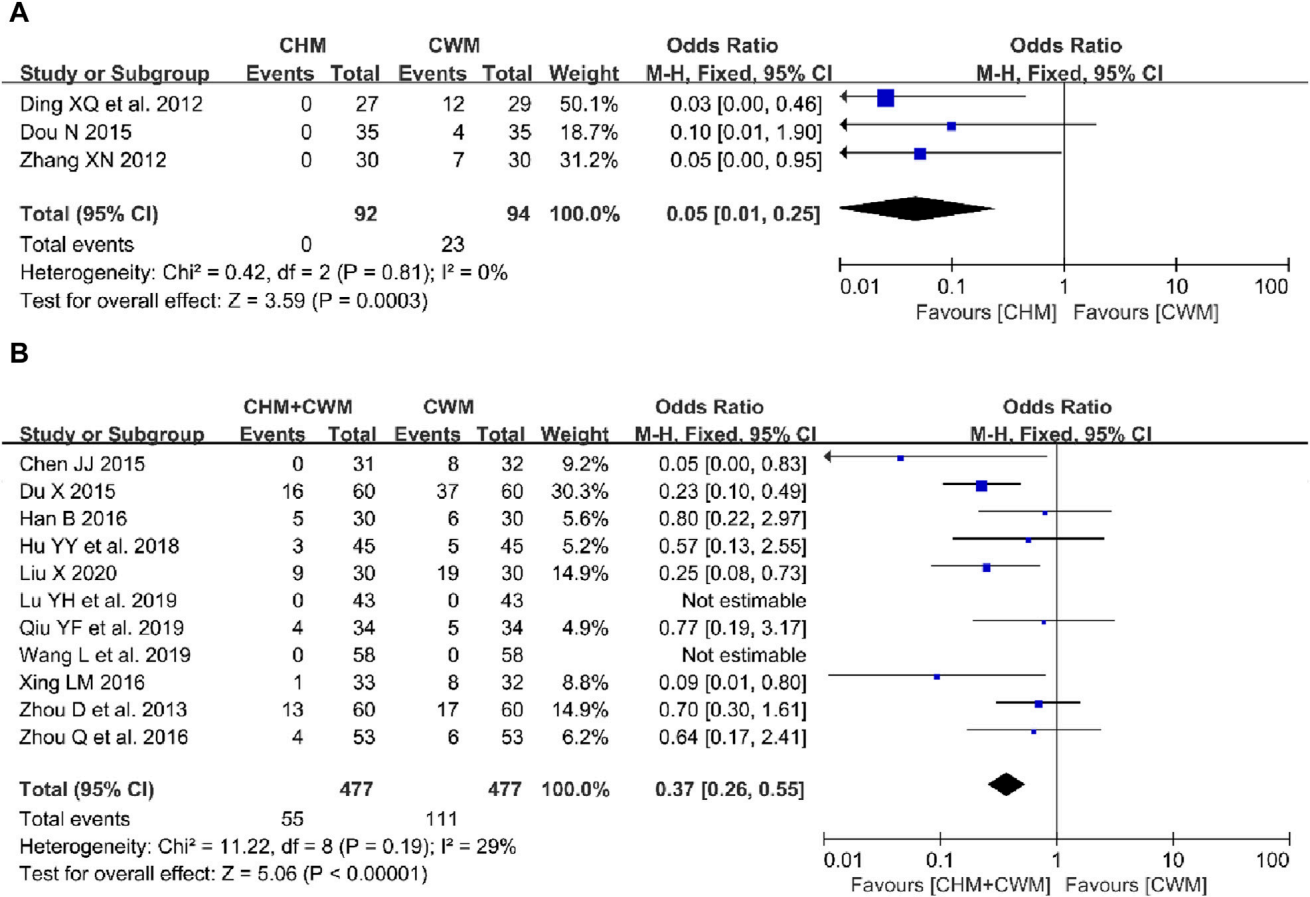
Forest plot illustrating the effect on adverse events of different intervention modes of CHM: **(A)** CHM Alone vs CWM, **(B)** CHM + CWM vs CWM.

Three studies (Ding and Shi, 2012; Zhang, 2012; Dou, 2015) encompassing 186 patients reported adverse effects in the CHM alone and the CWM group, displaying no heterogeneity across the studies. The incidence of adverse reactions was significantly lower in the CHM group compared to the CWM group (OR = 0.05; 95% CI: 0.01–0.25; *I* ^*2*^ = 0%, *p* = 0.0003; Figure 8A). Notably, adverse events such as perimenopausal symptoms (Ding and Shi, 2012; Zhang, 2012; Dou, 2015), androgenic reactions (Ding and Shi, 2012), and hepatic impairment (Ding and Shi, 2012; Zhang, 2012) were documented. Meta-analysis, as illustrated in Table 5, revealed a reduced incidence of perimenopausal symptoms (3 trials, n = 186; OR = 0.09; 95% CI: 0.02–0.50; *I* ^*2*^ = 0%, *p* = 0.004) and hepatic impairment (2 trials, n = 116; OR = 0.11; 95% CI: 0.01–0.94; *I* ^*2*^ = 0%, *p* = 0.04) in the CHM group compared to the CWM group. However, the difference in androgenic responses was not statistically significant (1 trial, n = 56; OR = 0.14; 95% CI: 0.01–2.80; *p* = 0.31).

**TABLE 5:** The effect of CHM intervention on the incidence of specific adverse events.

Outcome or subgroup	Studies	Patients	Heterogeneity		Effect measure	Or (95% CI)	*p*
			*p*	I^2^/%			
CHM Alone vs CWM							
Perimenopausal Symptoms	3 (Ding and Shi, 2012; Zhang, 2012; Dou, 2015)	186	0.98	0	Odds Ratio	0.09 [0.02, 0.50]	0.004
Androgenic Response	1 (Ding and Shi, 2012)	56	-	-	Odds Ratio	0.14 [0.01, 2.80]	0.31
Hepatic Function Impairment	2 (Ding and Shi, 2012; Zhang, 2012)	116	0.92	0	Odds Ratio	0.11 [0.01, 0.94]	0.04
CHM + CWM vs CWM							
Perimenopausal Symptoms	8 (Chen, 2015; Du, 2015; Han, 2016; Xing, 2016; Zhou, 2016; Hu and Y, 2018; Qiu and Wan, 2019; Liu, 2020)	632	0.73	0	Odds Ratio	0.36 [0.21, 0.61]	0.0001
Androgenic Response	3 (Zhou and Liu, 2013; Du, 2015; Zhou, 2016)	346	0.81	0	Odds Ratio	0.58 [0.20, 1.65]	0.31
Hepatic Function Impairment	4 (Zhou and Liu, 2013; Chen, 2015; Xing, 2016; Liu, 2020)	308	0.41	0	Odds Ratio	0.39 [0.17, 0.92]	0.03
Gastrointestinal Discomfort	5 (Zhou and Liu, 2013; Du, 2015; Zhou, 2016; Hu and Y, 2018; Qiu and Wan, 2019)	504	0.99	0	Odds Ratio	0.75 [0.36, 1.58]	0.45
Physical Pain	3 (Du, 2015; Hu and Y, 2018; Qiu and Wan, 2019)	278	0.87	0	Odds Ratio	0.63 [0.16, 2.45]	0.50
Allergic Reactions	2 (Du, 2015; Hu and Y, 2018)	210	0.93	0	Odds Ratio	0.37 [0.08, 1.63]	0.19

**Abbreviation:** CHM: chinese herbal medicine; CWM: conventional western medicine.

##### 3.5.6.2 CHM + CWM vs CWM

Eleven studies investigated adverse reactions in groups receiving either a combination of CHM with CWM or CWM alone (Zhou and Liu, 2013; Chen, 2015; Du, 2015; Han, 2016; Xing, 2016; Zhou, 2016; Hu and Y, 2018; Lu et al., 2019; Qiu and Wan, 2019; Wang and Bo, 2019; Liu 2020). A fixed-effects model meta-analysis, depicted in Figure 8B, showed that the combination therapy significantly reduced the overall incidence of adverse events compared to CWM alone (OR = 0.37; 95% CI: 0.26–0.55; *I* ^*2*^ = 29%, *p* < 0.00001). Specific adverse reactions reported included perimenopausal symptoms (Chen, 2015; Du, 2015; Han, 2016; Xing, 2016; Zhou, 2016; Hu and Y, 2018; Qiu and Wan, 2019; Liu, 2020), androgenic response (Zhou and Liu, 2013; Du, 2015; Zhou, 2016), gastrointestinal discomfort (Zhou and Liu, 2013; Du, 2015; Zhou, 2016; Hu and Y, 2018; Qiu and Wan, 2019), physical pain (Du, 2015; Hu and Y, 2018; Qiu and Wan, 2019), allergic reactions (Du, 2015; Hu and Y, 2018), and hepatic function impairment (Zhou and Liu, 2013; Chen, 2015; Xing, 2016; Liu, 2020). Further analysis presented in Table 5 revealed that patients in the combined therapy group experienced fewer perimenopausal symptoms (8 trials, n = 632; OR = 0.36; 95% CI: 0.21–0.61; *I* ^*2*^ = 0%, *p* = 0.0001) and hepatic function impairment (4 trials, n = 308; OR = 0.39; 95% CI: 0.17–0.92; *I* ^*2*^ = 0%, *p* = 0.03) than those receiving CWM only. However, no significant differences were observed in androgenic responses (3 trials, n = 346; OR = 0.58; 95% CI: 0.20–1.65; *I* ^*2*^ = 0%, *p* = 0.31), gastrointestinal discomfort (5 trials, n = 504; OR = 0.75; 95% CI: 0.36–1.58; *I* ^*2*^ = 0%, *p* = 0.45), physical pain (3 trials, n = 278; OR = 0.63; 95% CI: 0.16–2.45; *I* ^*2*^ = 0%, *p* = 0.50), and allergic reactions (2 trials, n = 210; OR = 0.37; 95% CI: 0.08–1.63; *I* ^*2*^ = 0%, *p* = 0.19) between the two groups.

### 3.6 Subgroup analysis and sensitivity analysis

#### 3.6.1 Recurrence rate

**FIGURE 9:**
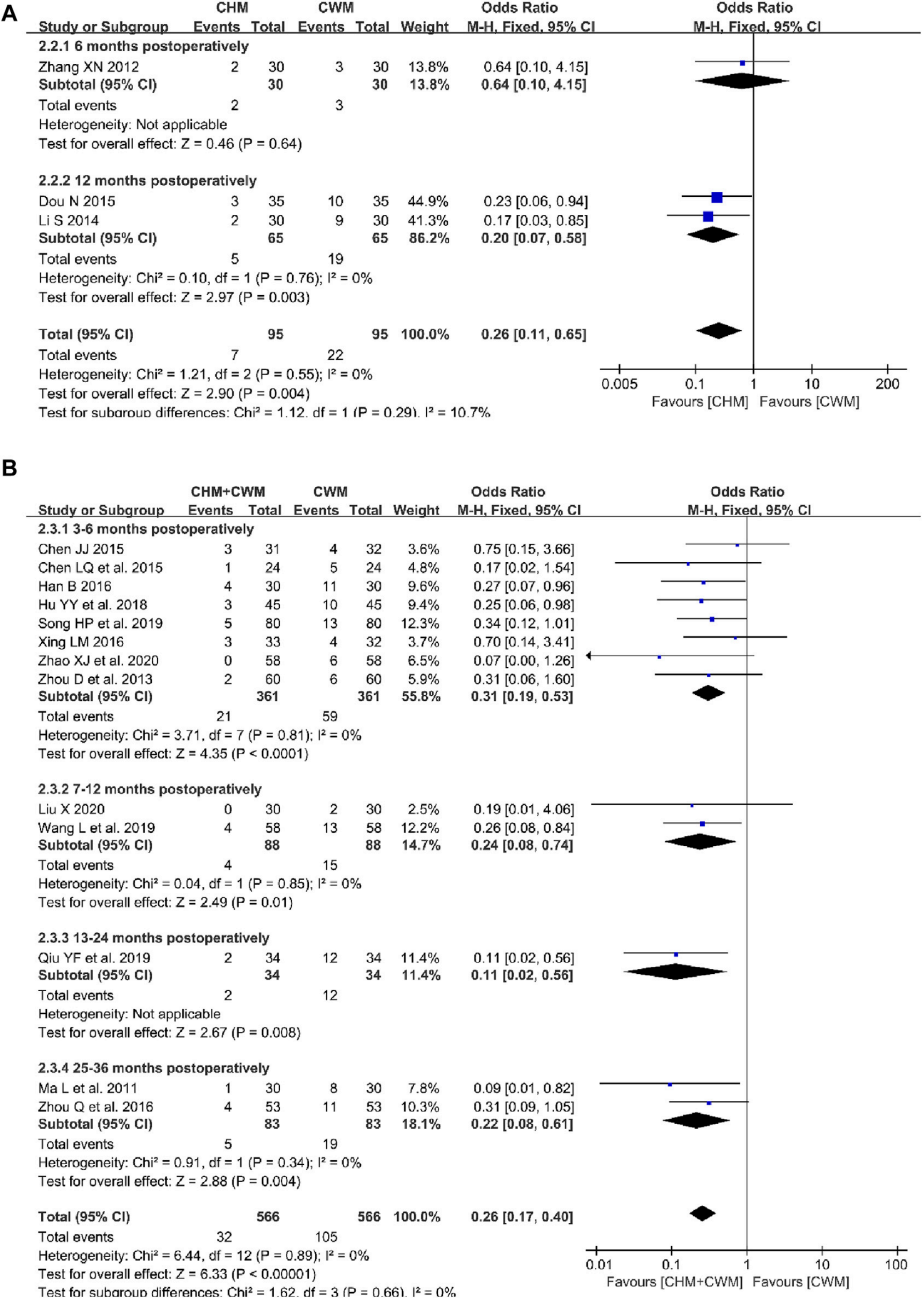
Forest plot illustrating the effect of CHM intervention modes on the recurrence rate of OEC during different follow-up periods: **(A)** CHM Alone vs CWM, **(B)** CHM + CWM vs CWM.

Based on the follow-up duration, we conducted a subgroup analysis on the postoperative recurrence rates in patients with OEC. As illustrated in Figure 9A, the recurrence rate in the group treated with CHM was lower than that in the CWM group at 12 months postoperatively (Li, 2014; Dou, 2015) (2 trials, n = 130; OR = 0.20; 95% CI: 0.07–0.58; *I* ^*2*^ = 0%, *p* = 0.003; Figure 9A), with no significant difference observed at 6 months postoperatively (Zhang, 2012) (1 trial, n = 60; OR = 0.64; 95% CI: 0.10–4.15; *p* = 0.64; Figure 9A). Meta-analysis revealed that patients receiving combined CHM and CWM treatment had significantly lower recurrence rates than those treated with CWM alone across several post-operative periods: 3–6 months (Zhou and Liu, 2013; Chen, 2015; Chen et al., 2015; Han, 2016; Xing, 2016; Hu and Y, 2018; Song et al., 2019; Zhao and Xiao, 2020) (8 trials, n = 722; OR = 0.31; 95% CI: 0.19–0.53; *I* ^*2*^ = 0%, *p* < 0.0001; Figure 9B), 7–12 months (2 trials, n = 176; OR = 0.24; 95% CI: 0.08–0.74; *I* ^*2*^ = 0%, *p* = 0.01; Figure 9B), 13–24 months (1 trial, n = 68; OR = 0.11; 95% CI: 0.02–0.56; *p* = 0.008; Figure 9B), and 25–36 months (2 trials, n = 166; OR = 0.22; 95% CI: 0.08–0.61; *I* ^*2*^ = 0%, *p* = 0.004; Figure 9B).

#### 3.6.2 VAS score

**FIGURE 10:**
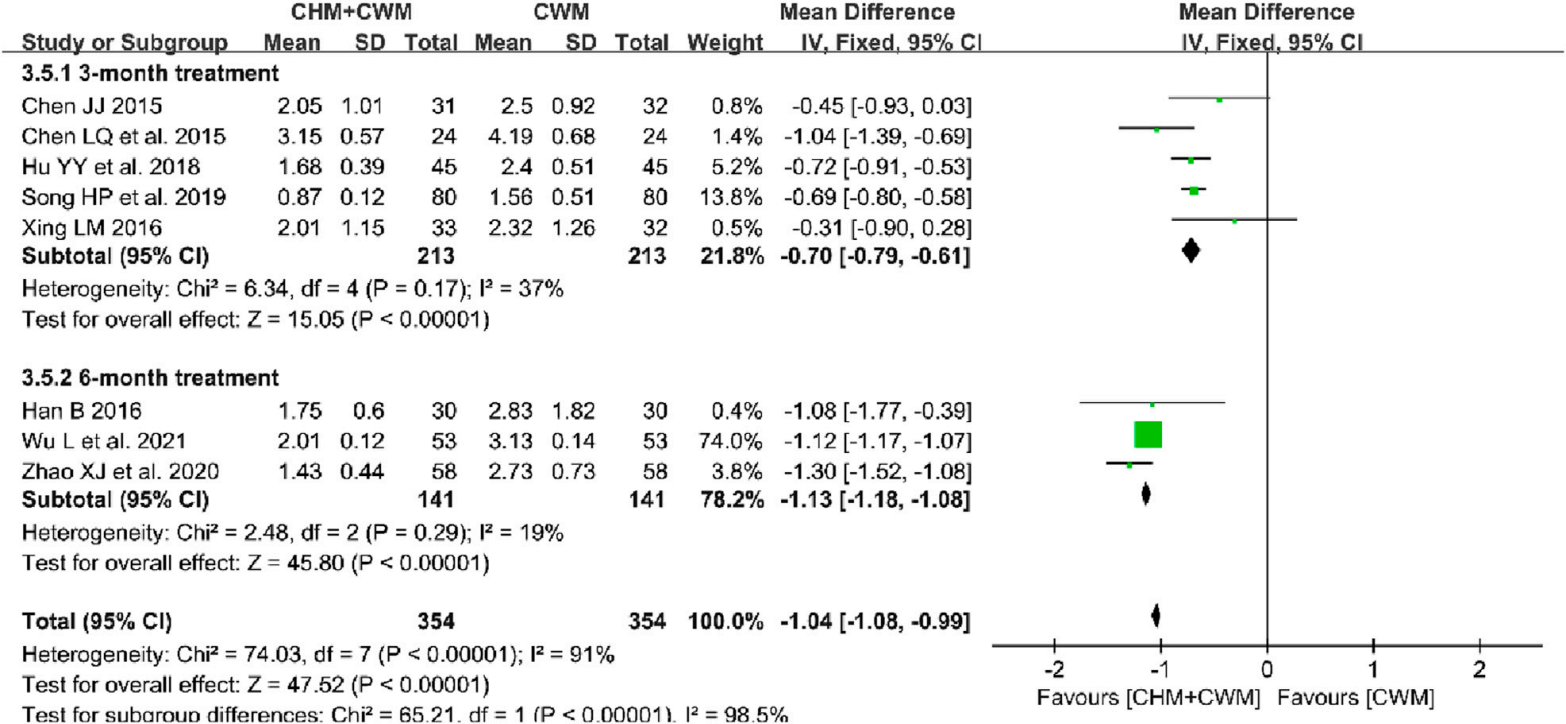
Forest plot depicting the effect of different durations of postoperative CHM combined with CWM treatment on reducing the VAS score.

A subgroup analysis of postoperative VAS scores in OEC patients was performed, categorized by treatment duration. Analysis revealed that a 6-month CHM and CWM treatment led to significantly lower VAS scores (Han, 2016; Zhao and Xiao, 2020; Wu and Deng, 2021) (3 trials, n = 282; MD = −1.13; 95% CI: -1.18 to −1.08; *p* < 0.00001) than a 3-month regimen (Chen, 2015; Chen et al., 2015; Xing, 2016; Hu and Y, 2018; Song et al., 2019) (5 trials, n = 426; MD = −0.70; 95% CI: -0.79 to −0.61; *p* < 0.00001) (Figure 10). Furthermore, the combination of CHM and CWM notably decreased VAS scores compared to CWM alone, regardless of the treatment’s duration (3 or 6 months). The results of this meta-analysis can be considered stable since no significant changes were noted in the leave-one-out sensitivity analysis.

#### 3.6.3 Serum level of CA125

**FIGURE 11:**
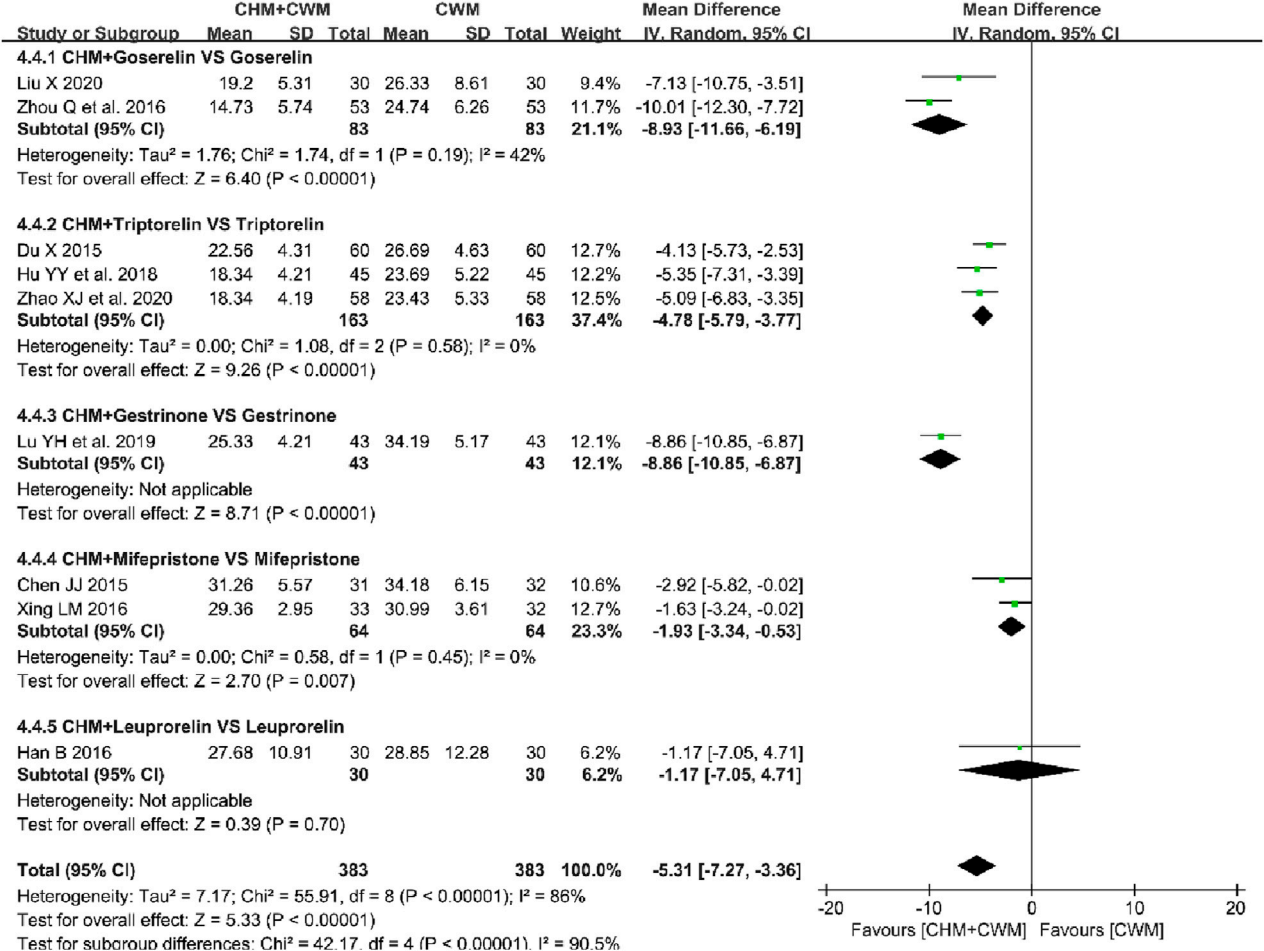
Forest plot illustrating the effect of different Western medications combined with CHM on the reduction of serum CA125 levels.

A subgroup analysis was conducted on postoperative serum CA125 levels in OEC patients treated with a CHM and CWM combination, targeting specific Western medications. Heterogeneity within subgroups was reduced. As shown in Figure 11, the meta-analysis results indicated a significant decrease in serum CA125 levels, following the combination of CHM with Goserelin (Zhou, 2016; Liu, 2020) (2 trials, n = 166; MD = −8.93; 95% CI: -11.66 to −6.19; *I* ^*2*^ = 42%, *p* < 0.00001), Triptorelin (Du, 2015; Hu and Y, 2018; Zhao and Xiao, 2020) (3 trials, n = 326; MD = −4.78; 95% CI: -5.79 to −3.77; *I* ^*2*^ = 0%, *p* < 0.00001), Gestrinone (Lu et al., 2019) (1 trial, n = 86; MD = −8.86; 95% CI: -10.85 to −6.87; *p* < 0.00001), and Mifepristone (Chen, 2015; Xing, 2016) (2 trials, n = 128; MD = −1.93; 95% CI: -3.34 to −0.53; *I* ^*2*^ = 0%, *p* = 0.007). However, no significant differences were found in serum CA125 levels between groups combining CHM with Leuprorelin and those using Leuprorelin alone (HAN, 2016) (1 trial, n = 60; MD = −1.17; 95% CI: -7.05 to 4.71; *p* = 0.70).

Besides, the meta-analysis of CA125 levels in the CHM alone *versus* CWM group exhibited significant heterogeneity (*I* ^*2*^ = 95%). However, due to the limited number of included articles (only 2), it was not possible to conduct subgroup analyses. Hence, based on sensitivity analysis, the original studies were examined. The results revealed that patients who received CHM treatment for a duration of 6 months exhibited significantly reduced serum CA125 levels compared to the control group (Dou, 2015) (MD = −16.09; 95% CI: -19.17 to −13.01; *p* < 0.00001). Nonetheless, following a 3-month CHM intervention, there was no discernible variation in the serum CA125 levels of the two groups (Zhang, 2012) (MD = −5.17; 95% CI: -12.07 to 1.73; *p* = 0.14). Differential treatment duration might be the primary source of heterogeneity.

### 3.7 Publication bias

**FIGURE 12:**
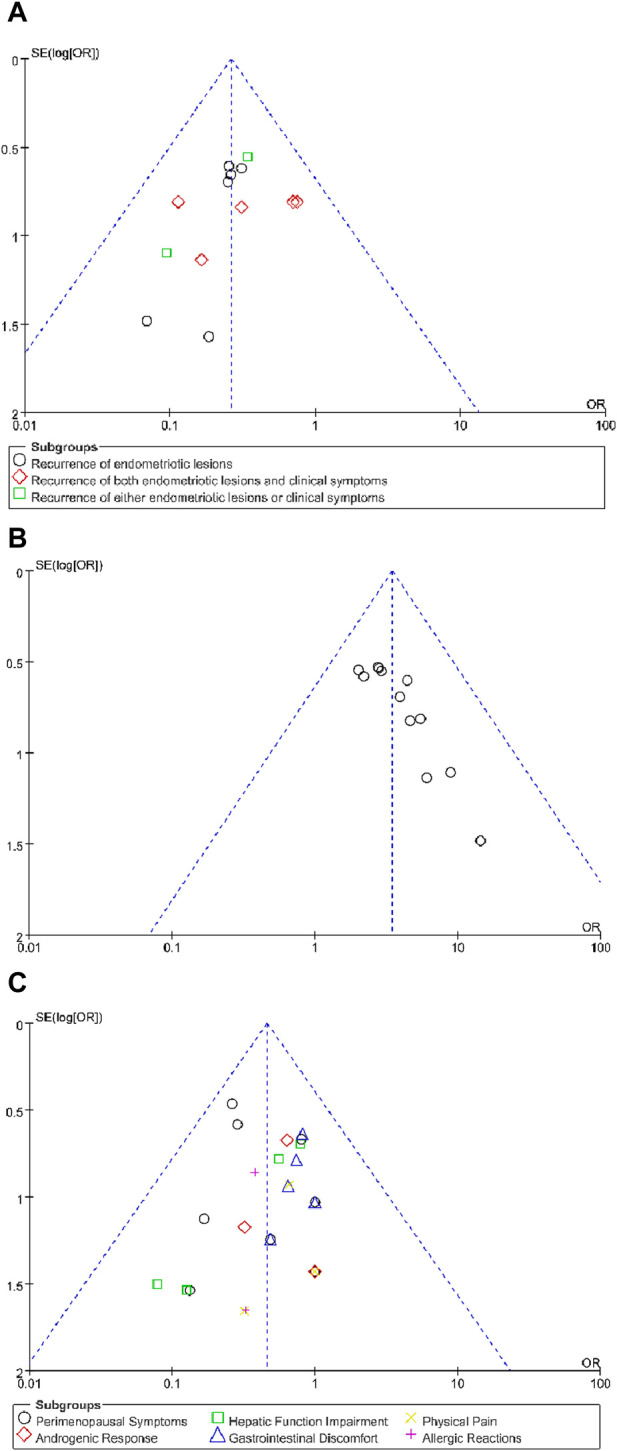
Funnel plot of relevant outcomes: **(A)** Recurrence rate, **(B)** Total clinical efficacy rate, **(C)** Adverse event.

Publication bias was detected through the funnel plot analysis of recurrence rate, total clinical efficacy rate and adverse event. The asymmetry suggested mild publication bias in the study (Figure 12).

### 3.8 GRADE evaluation of evidence quality

According to the GRADE standard (Guyatt et al., 2008b), GRADE profiler 3.2.2 was used to evaluate the quality of evidence for each outcome. Outcome indexes were classified into four grades of high quality, moderate quality, low quality and very low quality according to five aspects of research limitation, inconsistency, indirectness, imprecision, and publication bias. The evidence Profile with quality assessment and summary of findings were reported in Table 6.

**TABLE 6:** GRADE rating of the quality of each outcome.

Group	Outcomes	Anticipated absolute effects∗ (95% CI)		Relative effect (95% CI)	No of participants (studies)	Certainty of the evidence (GRADE)
		Risk with comparison	Risk with intervention			
**Intervention:** CHM Alone **Comparison:** Blank Control	Recurrence Rate	333 per 1,000	111 per 1,000 (48–242)	OR 0.25 (0.1–0.64)	126 (1 study)	⊕⊕⊝⊝ low^1,2^
	Total Clinical Efficacy Rate	825 per 1,000	952 per 1,000 (841–987)	OR 4.23 (1.12–15.99)	126 (1 study)	⊕⊕⊝⊝ low^1,2^
	Visual Analog Scale Score	-	MD 0.86 lower (1.01–0.71 lower)	-	126 (1 study)	⊕⊕⊝⊝ low^1,2^
	Pregnancy rate	127 per 1,000	365 per 1,000 (189–586)	OR 3.95 (1.6–9.74)	126 (1 study)	⊕⊕⊝⊝ low^1,2^
**Intervention:** CHM Alone **Comparison:** CWM	Recurrence Rate	232 per 1,000	73 per 1,000 (32–164)	OR 0.26 (0.11–0.65)	190 (3 studies)	⊕⊕⊝⊝ low^1,2^
	Total Clinical Efficacy Rate	723 per 1,000	885 per 1,000 (778–944)	OR 2.94 (1.34–6.43)	186 (3 studies)	⊕⊕⊝⊝ low^1,2^
	Visual Analog Scale Score	-	MD 0.16 lower (0.49 lower to 0.17 higher)	-	60 (1 study)	⊕⊕⊝⊝ low^1,2^
	CA125	-	MD 14.28 lower (17.09–11.47 lower)	-	130 (2 studies)	⊕⊝⊝⊝ very low^1,2,3^
	Pregnancy rate	373 per 1,000	663 per 1,000 (454–823)	OR 3.31 (1.4–7.83)	116 (2 studies)	⊕⊕⊝⊝ low^1,2^
	Adverse event rate	245 per 1,000	16 per 1,000 (3–75)	OR 0.05 (0.01–0.25)	186 (3 studies)	⊕⊕⊝⊝ low^1,2^
	Recurrence Rate (6 months postoperatively)	100 per 1,000	66 per 1,000 (11–316)	OR 0.64 (0.1–4.15)	60 (1 study)	⊕⊕⊝⊝ low^1,2^
	Recurrence Rate (12 months postoperatively)	292 per 1,000	76 per 1,000 (28–193)	OR 0.2 (0.07–0.58)	130 (2 studies)	⊕⊕⊝⊝ low^1,2^
	Adverse event rate (Perimenopausal Symptoms)	138 per 1,000	14 per 1,000 (3–74)	OR 0.09 (0.02–0.5)	186 (3 studies)	⊕⊕⊝⊝ low^1,2^
	Adverse event rate (Androgenic Response)	103 per 1,000	16 per 1,000 (1–243)	OR 0.14 (0.01–2.8)	56 (1 study)	⊕⊕⊝⊝ low^1,2^
	Adverse event rate (Hepatic Function Impairment)	119 per 1,000	15 per 1,000 (1–113)	OR 0.11 (0.01–0.94)	116 (2 studies)	⊕⊕⊝⊝ low^1,2^
**Intervention:** CHM + CWM **Comparison:** CWM	Recurrence Rate	186 per 1,000	56 per 1,000 (37–84)	OR 0.26 (0.17–0.4)	1,132 (13 studies)	⊕⊕⊕⊝ moderate^1^
	Total Clinical Efficacy Rate	803 per 1,000	933 per 1,000 (906–953)	OR 3.44 (2.37–5)	1,228 (12 studies)	⊕⊕⊕⊝ moderate^1^
	Visual Analog Scale Score	-	MD 1.04 lower (1.08–0.99 lower)	-	708 (8 studies)	⊕⊕⊝⊝ low^1,3^
	CA125	-	MD 5.1 lower (5.8–4.4 lower)	-	766 (9 studies)	⊕⊕⊝⊝ low^1,3^
	Pregnancy rate	139 per 1,000	326 per 1,000 (171–530)	OR 2.99 (1.28–6.98)	244 (3 studies)	⊕⊕⊝⊝ low^1,2^
	Adverse event rate	233 per 1,000	101 per 1,000 (73–143)	OR 0.37 (0.26–0.55)	954 (11 studies)	⊕⊕⊕⊝moderate^1^
	Recurrence Rate (3–6 months postoperatively)	163 per 1,000	57 per 1,000 (36–94)	OR 0.31 (0.19–0.53)	722 (8 studies)	⊕⊕⊕⊝moderate^1^
	Recurrence Rate (7–12 months postoperatively)	170 per 1,000	47 per 1,000 (16–132)	OR 0.24 (0.08–0.74)	176 (2 studies)	⊕⊕⊝⊝ low^1,2^
	Recurrence Rate (13–24 months postoperatively)	353 per 1,000	57 per 1,000 (11–234)	OR 0.11 (0.02–0.56)	68 (1 study)	⊕⊕⊝⊝ low^1,2^
	Recurrence Rate (25–36 months postoperatively)	229 per 1,000	61 per 1,000 (23–153)	OR 0.22 (0.08–0.61)	166 (2 studies)	⊕⊕⊝⊝ low^1,2^
	VAS Score (3-month treatment)	-	MD 0.7 lower (0.79–0.61 lower)	-	426 (5 studies)	⊕⊕⊕⊝moderate^1^
	VAS Score (6-month treatment)	-	MD 1.13 lower (1.18–1.08 lower)	-	282 (3 studies)	⊕⊕⊝⊝ low^1,2^
	CA125 (CHM + Goserelin VS Goserelin)	-	MD 8.93 lower (11.66–6.19 lower)	-	166 (2 studies)	⊕⊕⊝⊝ low^1,2^
	CA125 (CHM + Triptorelin VS Triptorelin)	-	MD 4.78 lower (5.79–3.77 lower)	-	326 (3 studies)	⊕⊕⊝⊝ low^1,2^
	CA125 (CHM + Gestrinone VS Gestrinone)	-	MD 8.86 lower (10.85–6.87 lower)	-	86 (1 study)	⊕⊕⊝⊝ low^1,2^
	CA125 (CHM + Mifepristone VS Mifepristone)	-	MD 1.93 lower (3.34–0.53 lower)	-	128 (2 studies)	⊕⊕⊝⊝ low^1,2^
	CA125 (CHM + Leuprorelin VS Leuprorelin)	-	MD 1.17 lower (7.05–4.71 lower)	-	60 (1 study)	⊕⊕⊝⊝ low^1,2^
	Adverse event rate (Perimenopausal Symptoms)	174 per 1,000	70 per 1,000 (42–114)	OR 0.36 (0.21–0.61)	632 (8 studies)	⊕⊕⊕⊝moderate^1^
	Adverse event rate (Androgenic Response)	58 per 1,000	34 per 1,000 (12–92)	OR 0.58 (0.20–1.65)	346 (3 studies)	⊕⊕⊕⊝moderate^1^
	Adverse event rate (Hepatic Function Impairment)	117 per 1,000	49 per 1,000 (22–109)	OR 0.39 (0.17–0.92)	308 (4 studies)	⊕⊕⊕⊝moderate^1^
	Adverse event rate (Gastrointestinal Discomfort)	67 per 1,000	51 per 1,000 (25–102)	OR 0.75 (0.36–1.58)	504 (5 studies)	⊕⊕⊕⊝moderate^1^
	Adverse event rate (Physical Pain)	36 per 1,000	23 per 1,000 (6–84)	OR 0.63 (0.16–2.45)	278 (3 studies)	⊕⊕⊝⊝ low^1,2^
	Adverse event rate (Allergic Reactions)	57 per 1,000	22 per 1,000 (5–90)	OR 0.37 (0.08–1.63)	210 (2 studies)	⊕⊕⊝⊝ low^1,2^

*The risk in the intervention group (and its 95% confidence interval) is based on the assumed risk in the comparator group and the relative effect of the intervention (and its 95% CI). CI: Confidence interval; MD: Mean difference; OR: Odds ratio. Factors of downgrade: 1 Unclear risk of detection bias, selection bias and performance bias; 2 Sample size is less than the optimal information size; 3 Significant statistical heterogeneity. GRADE Working Group grades of evidence: High quality: Further research is very unlikely to change our confidence in the estimate of effect. Moderate quality: Further research is likely to have an important impact on our confidence in the estimate of effect and may change the estimate. Low quality: Further research is very likely to have an important impact on our confidence in the estimate of effect and is likely to change the estimate. Very low quality: We are very uncertain about the estimate.

## 4 Discussion

OEC, a prevalent disorder in women of reproductive age, is known for its persistent nature and propensity for recurrence (Ceccaroni et al., 2019). Recently, some academics have incorporated the notion and framework of chronic disease management into the therapy of OEC, aiming to provide patients with comprehensive, uninterrupted, and proactive techniques for managing their condition (Falcone and Flyckt, 2018). Traditional Chinese Medicine (TCM) boasts a history spanning thousands of years. Recent studies have confirmed that CHM exerts therapeutic effects on OEC through multitarget mechanisms, minimizing adverse effects (Flower et al., 2012; Meresma et al., 2021; Chen et al., 2023; Zhao, 2023). To our knowledge, this is the first comprehensive systematic review and meta-analysis that integrates discussions on the principles of TCM treatment and laws of formula composition to assess the efficacy and safety of CHM for postoperative OEC in the English language.

### 4.1 Summary of evidence

#### 4.1.1 Efficacy

The primary results derived from the meta-analysis are as follows.

(**i**) Conservative surgery combined with CHM treatment may reduce the recurrence of endometriotic lesions, enhance the overall clinical efficacy and pregnancy rates, and alleviate postoperative pain.
(**ii**) In comparison to CWM alone, CHM alone may offer higher clinical efficacy and pregnancy rates, reduce endometriotic lesion recurrence, and lower serum CA125 levels, without a significant improvement in pain symptoms.
(**iii**) The combination of CHM and CWM, *versus* CWM alone, can significantly increase the overall clinical efficacy and pregnancy rates, while also reducing the recurrence of endometriotic lesions and clinical symptoms, lowering CA125 levels, and alleviating postoperative pain.
(**iv**) Concerning the primary outcome, recurrence rate, CHM intervention has demonstrated efficacy in reducing both endometriotic lesion and clinical symptom recurrence. This might be attributed to its regulatory effects on the body’s internal environment, immune enhancement, inflammation suppression, and angiogenesis inhibition (Meresman et al., 2021; Wu et al., 2022). Subgroup analysis revealed that CHM, compared to CWM, significantly lowered the 12-month postoperative recurrence rate in OEC patients. However, no significant difference was observed at the 6-month follow-up, suggesting the therapeutic effects of CHM may be more pronounced over a longer duration. This observation aligns with the holistic and gradual healing approach traditionally attributed to CHM, which may not only target the symptoms but also the underlying imbalances contributing to the disease’s recurrence (Zhao, 2023). Moreover, our analysis demonstrated that combining CHM with CWM could consistently reduce the recurrence rate across all follow-up intervals (3–36 months) compared to using CWM alone. This synergy suggested that CHM might enhance the efficacy of CWM, thereby offering a more sustainable and tolerable long-term management strategy for OEC.
(**v**) The effect of CHM on alleviating postoperative pain in patients with OEC was evaluated using the Visual Analogue Scale. Our research indicated that the use of CHM alone does not significantly outperform CWM in terms of pain improvement. However, it is important to note this conclusion is based on limited evidence, highlighting the need for further rigorous studies to fully explore the potential of CHM as an independent modality for postoperative pain relief. Conversely, our analysis suggested a potentially positive role for CHM as an adjunct to CWM in postoperative OEC management, particularly in offering more effective pain relief in long-term treatment. This not only reflects the comprehensive effects of CHM in regulating pelvic microcirculation and alleviating inflammatory infiltration (Gao Y. et al., 2022; Lin et al., 2022; Yue et al., 2022) but also underscores the significance of an integrated Chinese and Western medical treatment strategy in enhancing postoperative quality of life.
(**vi**) CA125 is recognized as a marker for ovarian epithelial cell tumors. Although it exhibits lower sensitivity and specificity, making it not the most reliable indicator for diagnosing endometriosis (Association, 2021), elevated levels of CA125 are associated with the staging and clinical types of endometriosis (Kovalak et al., 2023). It is commonly used in clinical settings as a monitoring indicator to assess the progression of OEC and the response to treatment (Foster, 2016; Hirsch et al., 2016; Liu, 2020; Zhao and Xiao, 2020). Therefore, in this study, we have included CA125 levels as a secondary outcome, supplementing it with clinical outcomes such as recurrence rate and VAS score, to facilitate a more comprehensive evaluation of the therapeutic effects of CHM in the long-term management of OEC. Our meta-analysis results indicated that CHM interventions could effectively reduce postoperative CA125 levels. Notably, in subgroup analyses, combinations of CHM with Goserelin, Triptorelin, Gestrinone, and Mifepristone treatments have shown significant effects in lowering CA125 levels. However, combining CHM with Leuprorelin did not exhibit a synergistic effect in reducing CA125 levels, necessitating further in-depth research to explore potential influencing factors on its efficacy. These results prompt further considerations for treatment choices, with detailed comparisons between different treatment groups aiding clinicians in formulating more precise treatment plans. Sensitivity analysis revealed that after 6 months of treatment, the CA125 levels in the CHM group were lower than those in CWM group, with no significant difference observed at 3 months of treatment. As OEC is a chronic condition characterized by a long course and a propensity for recurrence, it is advisable in clinical treatment to consider extending the medication duration appropriately based on the patient’s condition, to enhance clinical efficacy.

#### 4.1.2 Safety and adverse events

Regarding safety, previous meta-analyses focused on the overall adverse effect incidence rates of CHM treatment in postoperative OEC patients (Fan et al., 2022). In contrast, specific adverse reaction indicators could more accurately highlight TCM’s advantages in pattern differentiation and treatment. This study revealed that CHM, alone or combined with CWM, was superior in reducing the overall adverse event incidence and improving perimenopausal symptoms and liver function. CHM mimics sex hormone effects, activating the hypothalamic-pituitary-ovarian axis and enhancing local microcirculation to improve ovarian blood supply (Chen, 2018). Consequently, it improves ovarian function, addresses low estrogen levels, and alleviates symptoms like tidal fever, night sweats, sleep disturbances, and irregular vaginal bleeding in the perimenopausal period. Additionally, according to TCM theory, the liver, viewed as pivotal in women’s prenatal basis, plays a crucial role in physiological functioning. If the liver fails to govern the free flow of qi, it could lead to the occurrence of gynecological diseases. Therefore, it is important to emphasize the regulation of viscera and bowels postoperatively. Orderly transformation and qi flow may contribute to alleviating liver function impairment (Zhang et al., 2022).

### 4.2 Therapeutic principles and medication laws of CHM

#### 4.2.1 Therapeutic principles

This study conducted a summary and synthesis of the included literature, revealing that medical practitioners adhered to the guiding principles of TCM “treat disease before it arises” and “concept of holism” in the treatment of postoperative OEC. To achieve personalized therapy, they clinically based their approach on the patient’s constitution, supporting right and dispelling evil, and utilizing pattern identification as the basis for determining treatment. In my opinion, achievement of clinical efficacy is closely related to traditional medical experience and extensive use of a large number of classical Chinese herbal formulas. Although not recorded in TCM, it does not affect the understanding of TCM pathogenesis and the clinical treatment of OEC. As symptoms and signs including tongue coating and pulse are the basis of diagnosis and treatment in TCM, TCM syndrome and formulae syndrome rather than disease were focused accordingly. The following text will describe and discuss specific treatment principles from two perspectives.

(i) prevent disease before it arises, and expel pathogens to prevent recurrence

According to TCM theory, OEC are classified as “*zhēng jiă*” (concretions and conglomerations), with blood stasis being pivotal in their development (Zhao, 2023). While surgical intervention can address the pathological product of “static blood”, it does not eradicate the underlying factors leading to postoperative recurrence. Consequently, postoperative treatment in TCM prioritizes the removal of excess pathogens and emphasizes the enhancement of blood circulation and the resolution of stasis, as outlined in Table 2. Clinically, tailored to the individual’s constitution, employs flexible application of CHMs known for their efficacy in breaking stasis and invigorating blood, including *P. persica* (L.) Batsch (Taoren), *A. sinensis* (Oliv.) Diels (Danggui), and *S. miltiorrhiza* Bunge (Danshen) to prevent the recurrence of OEC.

(ii) Based on individual conditions, support right and dispel evil, aiming to adjust the body’s constitution to a natural balance.

Following OEC surgery, patients may experience diminished immune function, and inadequate postoperative care might lead to a deficiency of healthy qi, disrupting the normal circulation of qi and blood. This disruption fosters the regeneration of static blood, perpetuating a cycle of OEC recurrence. Therefore, the postoperative care should comply with the human body’s innate resistance to evil and the physiological characteristics of the internal organs. The treatment aimed to supplement deficiency and support right, and regulate the viscera and bowels (Table 2). Observing the natures, flavors, and channel entry by these herbs (Table 4), their combination demonstrated the following effects: a) harmonizing the warm and cold natures to balance yin and yang; b) acrid opening and bitter downbearing to regulate the movement of qi; c) the combination of acrid and sweet medicinals supports yang, nourishing qi and blood; d) incorporating the expulsion of pathogen within tonifying effects. As shown in Table 3, the tonifying CHMs primarily included *Cuscuta chinensis* Lam. (Tusizi), *Glycyrrhiza glabra* L. (Gancao), *Dipsacus asper* Wall. Ex DC. (Xuduan), *P. lactiflora* Pall. (Baishao), *Astragalus mongholicus* Bunge (Huangqi), focusing on blood supplementation and invigoration while boosting qi and warming yang, simultaneously nourishing the blood, and dispelling stasis. Additionally, postoperative medication primarily targeted the foot *jueyin* liver channel, followed by the hand *shaoyin* heart channel, the foot *taiyin* spleen channel, and the foot *shaoyin* kidney channel (Table 4). Furthermore, recognizing the emotional challenges faced by patients post-surgery, the treatment also aims to soothe the liver, nourish the heart, fortify the spleen, and reinforce the kidney. Achieving equilibrium in *zang-fu* functions ensures a harmonious flow of qi and blood, fostering a balanced constitution and natural elimination of blood stasis.

#### 4.2.2 Description of high-frequency CHM

**FIGURE 13:**
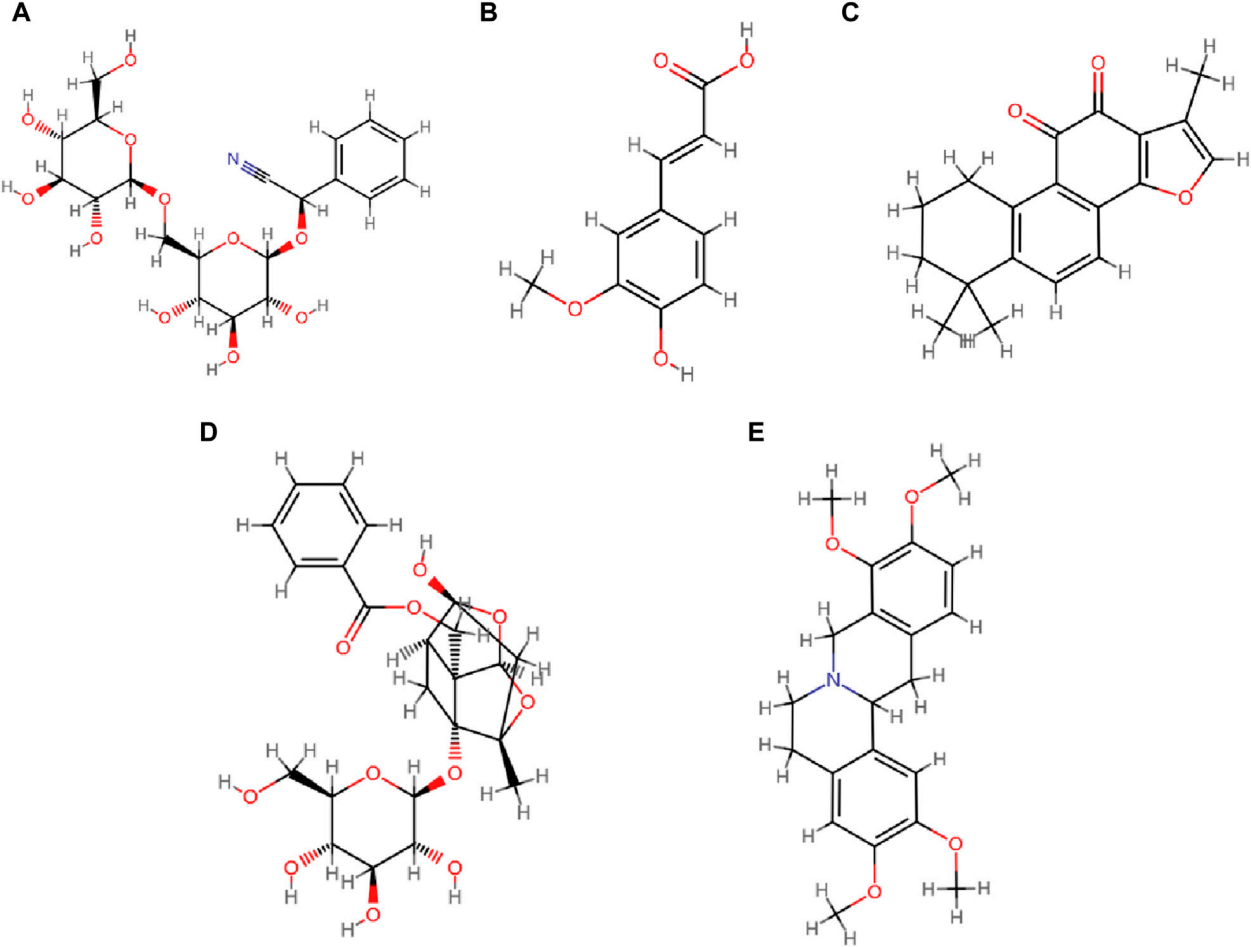
Chemical structures of the main active metabolites of most frequently used CHM for postoperative treatment of OEC: **(A)** Amygdalin, **(B)** Ferulic acid, **(C)** Tanshinone IIA, **(D)** Paeoniflorin, **(E)** Tetrahydropalmatine.

The most frequently used CHMs were also analyzed in this study. Among the top five, *P. persica* (L.) Batsch (Taoren) possesses a bitter flavor, a neutral nature. It targets the hand *shaoyin* heart, foot *jueyin* liver, and hand *yangming* large intestine channels, exhibiting functions of invigorating blood and dissolving stasis. Its active metabolite, Amygdalin (Figure 13A), exhibits antioxidative, anti-tumor, anti-inflammatory properties, immune modulation, and analgesic effects (Barakat et al., 2022). Research has shown Amygdalin’s potential in triggering apoptosis in tumor cells and causing cell cycle arrest (Saleem et al., 2018).

*Angelica sinensis* (Oliv.) Diels (Danggui) is recognized for its sweet, acrid taste and warm nature, interacting with the foot *jueyin* liver, hand *shaoyin* heart, and the foot *taiyin* spleen channels. It aids in blood supplement and invigoration, menstrual regulation, and pain alleviation. Ferulic acid (Figure 13B), the active metabolite of Danggui, offers vascular endothelial protection through the ERK1/2 and NO/ET-1 pathways and exhibits anti-fibrosis properties via the TGF-β/Smad and MMPs/TIMPs systems (Li et al., 2021; Li et al., 2022).

*Salvia miltiorrhiza* Bunge (Danshen) has a bitter flavor and a slightly cold nature, entering the hand *shaoyin* heart channel and the foot *jueyin* liver channel. Its functions involve invigorating blood, dissolving stasis, regulating menstruation, and calming the mind, making it a crucial botanical drug in gynecology. Its principal active metabolite, tanshinone IIA (Figure 13C), plays a pivotal role in mitigating adhesion, invasion, and angiogenesis, thereby improving the pathological morphology of ectopic endometrium and inhibiting the formation of endometriotic lesions (Chen and Gong, 2020; Zhang et al., 2023).

*Paeonia lactiflora* Pall. (Chishao) is utilized to alleviate heat, cool the blood, and eliminate blood stasis, addressing conditions such as concretions, conglomerations, and amenorrhea effectively. Its active metabolite, paeoniflorin (Figure 13D), exhibits anti-inflammatory, antioxidant, immunomodulatory, and anti-tumor properties (Zhang and Wei, 2020; Wang Xz. et al., 2022). Furthermore, research suggests that paeoniflorin could regulate the metabolic expression of various pathways in a rat model of endometriosis (Wu et al., 2019).

*Corydalis yanhusuo* W.T.Wang (Yanhusuo) is widely used to alleviate general body pain. It exhibits significant anti-inflammatory, analgesic, neuroprotective, and antitumor properties, attributed to tetrahydropalmatine as its active metabolite (Figure 13E). A combination of tetrahydropalmatine, ferulic acid, and ligustrazine has been shown to inhibit epithelial-mesenchymal transition through the Wnt/β-catenin pathway (Zhang et al., 2021), exerting an anti-proliferative effect on endometriosis via modulation of the Notch pathway (Dai et al., 2023).

It is noteworthy that, oxidative stress, a negative effect stemming from the imbalance of oxidation system in the body, plays a significant role in apoptosis, aging, and the onset of various diseases (Reuter et al., 2010; Poprac et al., 2017; Hajam et al., 2022). OEC is intricately linked to oxidative damage, both in terms of its initiation and development (Matsuzaki and Schubert, 2010; Hayasti et al., 2020; Koninckx et al., 2021). Plant polyphenols, including flavonoids, tannins, phenolic acids, etc., have the ability to scavenge free radicals within the body, combat lipid oxidation, delay organismal aging (Yahfoufi et al., 2018; Maleki et al., 2019; Nani et al., 2021), and exhibit anti-inflammatory, anti-tumor, and analgesic properties (Arablou and Kolahdouz-Mohammadi, 2018; Liu et al., 2022). Numerous CHMs and natural metabolites, including the five botanical drugs previously mentioned, possess certain antioxidant, anti-tumor, anti-inflammatory, and anti-aging effects. These attributes underline the potential of CHMs in the multifaceted and comprehensive treatment of OEC.

### 4.3 Limitations and future perspectives

This review acknowledges several limitations that warrant consideration. First, suboptimal methodological design is a common issue in most included trials, demanding a careful explanation of our results. Second, the absence of sample size estimation in many studies undermines the precision of results, particularly in smaller-scale research. Third, the omission of OEC staging information in some studies may influence the aggregated results regarding OEC recurrence. Finally, the GRADE evaluation for various outcomes was of moderate to low quality, indicating constraints in stability and reliability of conclusions.

To address these limitations and improve the quality of future research, the following strategies are recommended. First, significant drawbacks regarding the sequence generation of randomization, concealment of allocation, reporting on blinding, attrition, and pre-estimation of sample size should be considered to minimize potential biases. Second, adopting a multicenter, large-sample research design is advisable to ensure the reliability and generalizability of results. Third, a detailed analysis of variables such as surgical techniques, patient age, severity of condition, and OEC staging could yield deeper insights. Fourth, integrating various types of real-world studies could be considered to enhance the evaluation of the therapeutic effects of CHM. Finally, future studies could further identify the specific components or formulations of CHM responsible for clinical benefits and elucidate the mechanisms of their synergistic effects with CWM. This would lay the groundwork for more personalized and effective treatment paradigms, integrating the best of traditional and modern medicine.

## 5 Conclusion

In general, this systematic review and meta-analysis suggested that CHM may be beneficial for the treatment of postoperative OEC in reducing recurrence, improving clinical efficacy, and decreasing side effects. CHM could serve as a potential candidate or supplemental therapy for postoperative OEC in the battle for long-term management. However, considering the limitations of existing studies, further large-scale, high-quality, and rigorously designed trials are warranted to substantiate the conclusions.

## Data Availability

The original contributions presented in the study are included in the article/Supplementary Material, further inquiries can be directed to the corresponding author.

## Supplementary material

SUPPLEMENTARY APPENDIX S1 List of PRISMA 2020 statements.

SUPPLEMENTARY APPENDIX S2 Search strategies for all the electronic databases.

SUPPLEMENTARY APPENDIX S3 Components of CHM in the included studies.
