# Supplementary material for: Exploring the role of Chinese herbal medicine in the long-term management of postoperative ovarian endometriotic cysts: a systematic review and meta-analysis
Source: Front Pharmacol. 2024 Jun 7;15:1376037. doi: 10.3389/fphar.2024.1376037 (PMC11190181; doi:10.3389/fphar.2024.1376037)
Supplement: Supplementary file 1 [file Table1.docx]

| **Supplementary Appendix 1** | | | |
| --- | --- | --- | --- |
| **Section and Topic** | **Item #** | **Checklist item** | **Location where item is reported** |
| **TITLE** | | |  |
| Title | 1 | The report is identified as a systematic review. |  |
| **ABSTRACT** | | |  |
| Abstract | 2 | The abstract provides a comprehensive summary of the background, methods, results, and conclusions. |  |
| **INTRODUCTION** | | |  |
| Rationale | 3 | Described in the introduction. |  |
| Objectives | 4 | This study has updated the relevant literature, conducted a comprehensive systematic review of RCTs to evaluate the current clinical evidence on CHM for postoperative OEC. Additionally, it provided a summarizing analysis of medication characteristics and treatment principles, aiming to offer assistance for clinical medication. |  |
| **METHODS** | | |  |
| Eligibility criteria | 5 | Described in the “2.1. Eligibility criteria” |  |
| Information sources | 6 | The search was conducted using PubMed, Web of Science, Cochrane Library, EMBASE, Chinese National Knowledge Infrastructure (CNKI), VIP Information Database (VIP), Chinese Biomedical Literature Database (CBM), and Wanfang Database from inception up to November 01, 2023. |  |
| Search strategy | 7 | Described in the “2.2. Literature search” | Supplementary Appendix 2 |
| Selection process | 8 | Described in the “2.3. Study selection and data extraction” |  |
| Data collection process | 9 | Described in the “2.3. Study selection and data extraction” |  |
| Data items | 10a | Described in the “2.3. Study selection and data extraction” |  |
|  | 10b | Described in the “2.3. Study selection and data extraction” |  |
| Study risk of bias assessment | 11 | Described in the “2.4. Assessment of methodological quality” |  |
| Effect measures | 12 | Mean difference or odds ratio was used in the synthesis or presentation of results. |  |
| Synthesis methods | 13a | Described in the “2.5. Data analysis” |  |
|  | 13b | Described in the “2.5. Data analysis” |  |
|  | 13c | Described in the “2.5. Data analysis” |  |
|  | 13d | Described in the “2.5. Data analysis” |  |
|  | 13e | Described in the “2.5. Data analysis” |  |
|  | 13f | Described in the “2.5. Data analysis” |  |
| Reporting bias assessment | 14 | Homogeneity across trails was evaluated using the I^2^ statistics. |  |
| Certainty assessment | 15 | We applied a fixed-effect model to assess treatment effects. A p-value < 0.05 was considered statistically significant. Publication bias was illustrated using a funnel plot. |  |
| **RESULTS** | | |  |
| Study selection | 16a | The screening process is shown in Figure 1 |  |
|  | 16b | Duplicate publication, wrong randomization, wrong intervention, not relevant to outcome measures, incomplete data, participants did not meet the inclusion criteria. |  |
| Study characteristics | 17 | Described in the “3.2. Characteristics of Included Trials” |  |
| Risk of bias in studies | 18 | Described in the “3.3. Assessment of Methodological Quality” |  |
| Results of individual studies | 19 | Described in the “3.5. Efficacy Assessment” |  |
| Results of syntheses | 20a | Quality assessment |  |
|  | 20b | Described in the “3.5. Efficacy Assessment” |  |
|  | 20c | Described in the “3.6. Subgroup Analysis and Sensitivity Analysis” |  |
|  | 20d | Described in the “3.6. Subgroup Analysis and Sensitivity Analysis” |  |
| Reporting biases | 21 | Described in the “3.7. Publication Bias” |  |
| Certainty of evidence | 22 | Described in the “3.7. Publication Bias” |  |
| **DISCUSSION** | | |  |
| Discussion | 23a | Described in the “4.1. Summary of Evidence” |  |
|  | 23b | Described in the “4.3. Limitations and Future Perspectives” |  |
|  | 23c | Described in the discussion. |  |
|  | 23d | Described in the “4.3. Limitations and Future Perspectives” |  |
| **OTHER INFORMATION** | | |  |
| Registration and protocol | 24a | All included studies were not registered in China. |  |
|  | 24b | Indicate where the review protocol can be accessed, or state that a protocol was not prepared. |  |
|  | 24c | Describe and explain any amendments to information provided at registration or in the protocol. |  |
| Support | 25 | Described in the Funding. |  |
| Competing interests | 26 | Declare any competing interests of review authors. |  |
| Availability of data, code and other materials | 27 | Report which of the following are publicly available and where they can be found: template data collection forms; data extracted from included studies; data used for all analyses; analytic code; any other materials used in the review. |  |

*From:*  Page MJ, McKenzie JE, Bossuyt PM, Boutron I, Hoffmann TC, Mulrow CD, et al. The PRISMA 2020 statement: an updated guideline for reporting systematic reviews. BMJ 2021;372, n71. doi: 10.1136/bmj.n71
