# Supplementary material for: Exploring the role of Chinese herbal medicine in the long-term management of postoperative ovarian endometriotic cysts: a systematic review and meta-analysis
Source: Front Pharmacol. 2024 Jun 7;15:1376037. doi: 10.3389/fphar.2024.1376037 (PMC11190181; doi:10.3389/fphar.2024.1376037)
Supplement: Supplementary file 2 [file Table3.docx]

**Supplementary Appendix 3**

**Components of CHM in the included studies**

| **References** |  | **Herbal** **formula** |  | **Ingredients** |
| --- | --- | --- | --- | --- |
| Tong QL et al. 2023 |  | *Fufang Xuanju* capsules |  | Yinyanghuo (*Epimedium sagittatum* (Siebold & Zucc.) Maxim.); Gouqi (*Lycium barbarum* L.); Shechuangzi (*Cnidium monnieri* (L.) Cusson). |
| Wu L et al. 2021 |  | *Cinnamon Twig and Poria* pills |  | Guizhi (*Neolitsea cassia* (L.) Kosterm.); Fuling  (*Carapichea ipecacuanha* (Brot.) L.Andersson); Mudanpi  (*Paeonia × suffruticosa* Andrews); Chishao (*Paeonia*  *lactiflora* Pall.); Taoren (*Prunus persica* (L.) Batsch). |
| Zhao XJ et al. 2020 |  | *Yishen Shugan* decoction |  | Shanzhuyu (*Cornus officinalis* Siebold & Zucc.) 20g; Bajitian (*Gynochthodes officinalis* (F.C.How) Razafim. & B.Bremer) 20g; Yinyanghuo (*Epimedium sagittatum* (Siebold & Zucc.) 20g; Gouji (*Cibotium barometz* (L.) J.Sm.)20g; Fupenzi (*Rubus chingii* Hu)20g; Duzhong (*Eucommia ulmoides* Oliv.) 15g; Chaihu (*Bupleurum chinense* DC.)15g; Yujin (*Curcuma longa* L.)15g; Xiangfu (*Cyperus rotundus* L.)15g; Lizhihe (*Litchi chinensis* Sonn.)10g; Mopancao (*Abutilon indicum* (L.) Sweet)10g. |
| Liu X 2020 |  | *Huayu Xiaozheng* decoction |  | Chishao (*Paeonia lactiflora* Pall.)15g; Bajitian  (*Gynochthodes officinalis* (F.C.How) Razafim. & B.Bremer) 10g; Sangjisheng (*Taxillus chinensis* (DC.) Danser) 20g; Tusizi (*Cuscuta chinensis* Lam.) 20g; Gancao (*Glycyrrhiza glabra* L.) 5g; Sanleng(*Sparganium stoloniferum* (Buch.-Ham. ex Graebn.) Buch.-Ham. ex Juz.) 10g; Ezhu (*Curcuma longa* L.) 10g; Tubiechong (*Eupolyphaga sinensis* Walker) 10g; Lulutong (*Liquidambar formosana* Hance) 10g; Lingzhi (*Ganoderma lucidum* (Curtis) P.Karst.) 10g; Hongjingtian (*Rhodiola crenulata* (Hook.f. & Thomson) H.Ohba) 10g; Tubeimu (*Bolbostemma paniculatum* (Maxim.) Franquet) 10g; Guijianyu (*Euonymus alatus* (Thunb.) Siebold) 10g; Tufuling (*Smilax glabra* Roxb.) 10g; Danggui (*Angelica sinensis* (Oliv.) Diels) 15g; Jineijin (*Gallus gallus domesticus* Brisson) 10g. |
| Qiu YF et al. 2019 |  | *Wenshen Xiaozheng* decoction |  | Moyao (*Commiphora myrrha* (T.Nees) Engl.) 10g; Wulingzhi (*Trogopterori Faeces*) 10g; Danshen (*Salvia miltiorrhiza* Bunge) 20g; Yanhusuo (*Corydalis yanhusuo* (Y.H.Chou & Chun C.Hsu) W.T.Wang ex Z.Y.Su & C.Y.Wu) 10g; Xuduan (*Dipsacus asper* Wall. Ex DC.) 10g; Huangqi (*Astragalus mongholicus* Bunge) 15g; Chuanlianzi (*Melia azedarach* L.) 10g; Xuejie (*Calamus draco* Willd.) 2g. |
| Wang L et al. 2019 |  | *Xiaojin* capsules |  | Danggui (*Angelica sinensis* (Oliv.) Diels); Shexiang (*Moschus berezovskii* Flerov); Mubiezi (*Momordica cochinchinensis* (Lour.) Spreng.); Zhicaowu (*Aconitum kusnezoffii* Rchb.); Fengxiangzhi (*Liquidambar formosana* Hance); Ruxiang (*Boswellia sacra* Flück.); Moyao (*Commiphora myrrha* (T.Nees) Engl.); Wulingzhi (*Trogopterori Faeces*); Dilong (*Pheretima aspergillum* (E.Perrier)); Xiangmo (Atramentum). |
| Song HP et al. 2019 |  | *Kuntai* capsules |  | Fuling (*Carapichea ipecacuanha* (Brot.) L.Andersson);  Shudi (*Rehmannia glutinosa* (Gaertn.) DC.);  Huanglian (*Coptis chinensis* Franch.); Baishao (*Paeonia*  *lactiflora* Pall.); Huangqin (*Scutellaria baicalensis*  Georgi); Ejiao (*Equus asinus* L.). |
| Lu YH et al. 2019 |  | *Guizhi Fuling* capsules |  | Guizhi (*Neolitsea cassia* (L.) Kosterm.); Fuling  (*Carapichea ipecacuanha* (Brot.) L.Andersson); Mudanpi  (*Paeonia × suffruticosa* Andrews); Chishao (*Paeonia*  *lactiflora* Pall.); Taoren (*Prunus persica* (L.) Batsch). |
| Hu YY et al. 2018 |  | *Dan’e Fukang* decocted extract |  | Chishao (*Paeonia lactiflora* Pall.); Chaihu (*Bupleurum*  *chinense* DC.); Xiangfu (*Cyperus rotundus* L.); Gancao  (*Glycyrrhiza glabra* L.); Sanleng (*Sparganium*  *stoloniferum* (Buch.-Ham. ex Graebn.) Buch.-Ham. Ex  Juz.); Ezhu (*Curcuma longa* L.); Danggui (*Angelica*  *sinensis* (Oliv.) Diels); Danshen (*Salvia miltiorrhiza*  Bunge); Sanqi (*Panax notoginseng* (Burkill) F.H.Chen);  Yanhusuo (*Corydalis yanhusuo* (Y.H.Chou & Chun  C.Hsu) W.T.Wang ex Z.Y.Su & C.Y.Wu). |
| Chen M et al. 2018 |  | *Neiyi* decoction |  | Taoren (*Prunus persica* (L.) Batsch) 20g; Tubiechong  (*Eupolyphaga sinensis* Walker)10g; Wulingzhi  (*Trogopterori Faeces*) 15g; Danshen (*Salvia miltiorrhiza*  Bunge) 20g; Yanhusuo (*Corydalis yanhusuo* (Y.H.Chou  & Chun C.Hsu) W.T.Wang ex Z.Y.Su & C.Y.Wu) 15g;  Wumei (*Prunus mume* (Siebold) Siebold & Zucc.) 25g;  Muli (*Ostrea gigas* Thunberg) 20g; Puhuang (*Typha*  *angustifolia* L.) 15g; Yimucao (*Leonurus japonicus*  Houtt.) 15g; Haizao (*Sargassum pallidum* (Turn.)  C.Ag.)15g; Chuanxiong (*Conioselinum anthriscoides*  *'Chuanxiong'*) 15g; Zhebeimu (*Fritillaria thunbergia*  Miq.) 10g; Xuduan (*Dipsacus asper* Wall. Ex DC.)15g. |
| Zhou Q et al. 2016 |  | *Fuzheng Xiaoyi* decoction |  | Duzhong (*Eucommia ulmoides* Oliv.) 15g; Baishao  (*Paeonia lactiflora* Pall.)10g; Zhebeimu (*Fritillaria*  *thunbergia* Miq.) 15g; Shanzha (*Crataegus monogyna*  Jacq.) 25g; Huangqi (*Astragalus mongholicus*  Bunge)15g; Lujiaoshuang (*Cervi Cornu Degelatinatum*)  10g; Xuejie (*Calamus draco* Willd.) 5g; Banbianlian  (*Lobelia chinensis* Lour.) 25g; Zaojiaoci (*Gleditsia*  *sinensis* Lam.) 10g; Daji (*Cirsium japonicum* DC.) 10g;  Xiaoji (*Cirsium arvense var. arvense*) 10g. |
| Xing LM 2016 |  | Empirical formula |  | Mudanpi (*Paeonia × suffruticosa* Andrews) 6g; Chishao  (*Paeonia lactiflora* Pall.) 10g; Xiangfu (*Cyperus*  *rotundus* L.)10g; Tusizi (*Cuscuta chinensis* Lam.) 10g;  Gancao (*Glycyrrhiza glabra* L.) 6g; Sanleng(*Sparganium*  *stoloniferum* (Buch.-Ham. ex Graebn.) Buch.-Ham. Ex  Juz.) 10g; Ezhu (*Curcuma longa* L.) 10g; Danggui  (*Angelica sinensis* (Oliv.) Diels) 10g; Shudi (*Rehmannia*  *glutinosa* (Gaertn.) DC.) 20g; Jixueteng (*Spatholobus*  *suberectus* Dunn) 15g; Honghua (*Carthamus tinctorius*  L.) 6g; Chenpi (*Citrus reticulata* Blanco) 6g. |
| Han B 2016 |  | *Turtle Shell* decocted pills |  | Guizhi (*Neolitsea cassia* (L.) Kosterm.); Mudanpi  (*Paeonia × suffruticosa* Andrews); Taoren (*Prunus*  *persica* (L.) Batsch); Chaihu (*Bupleurum chinense* DC.);  Tubiechong (*Eupolyphaga sinensis* Walker); Baishao  (*Paeonia lactiflora* Pall.); Huangqin (*Scutellaria*  *baicalensis* Georgi); Ejiao (*Equus asinus* L.); Danshen  (*Salvia miltiorrhiza* Bunge); Biejia (*Trionyx sinensis*  Wiegmann); Fengfang (*Polistes olivaceous* (DeGeer));  Qianglang (*Catharsius molossus* Linnaeus); Xiaoshi  (Nitrum); Banxia (*Pinellia ternata* (Thunb.) Makino);  Ganjiang (*Zingiber officinale* Roscoe); Houpo (*Magnolia*  *officinalis* Rehder &E.H.Wilson); Shegan (*Iris domestica*  (L.) Goldblatt & Mabb.); Dahuang (*Rheum officinale*  Baill.); Lingxiaohua (*Campsis grandiflora* (Thunb.)  K.Schum.); Tinglizi (*Descurainia sophia* (L.) Webb ex  Prantl); Shiwei (*Pyrrosia lingua* (Thunb.) Farw.); Qumai  (*Dianthus chinensis* L.). |
| Du X 2015 |  | *Neiyi* decoction |  | Taoren (*Prunus persica* (L.) Batsch) 20g; Tubiechong  (*Eupolyphaga sinensis* Walker) 10g; Wulingzhi  (*Trogopterori Faeces*) 15g; Danshen (*Salvia miltiorrhiza*  Bunge) 20g; Yanhusuo (*Corydalis yanhusuo* (Y.H.Chou  & Chun C.Hsu) W.T.Wang ex Z.Y.Su & C.Y.Wu) 15g;  Wumei (*Prunus mume* (Siebold) Siebold & Zucc.) 25g;  Muli (*Ostrea gigas* Thunberg) 20g; Puhuang (*Typha*  *angustifolia* L.) 15g; Wuyao (*Lindera aggregata* (Sims)  Kosterm.)15g; Yimucao (*Leonurus japonicus* Houtt.)  15g; Haizao (*Sargassum pallidum* (Turn.) C.Ag.)15g;  Chuanxiong (*Conioselinum anthriscoides 'Chuanxiong'*)  15g; Zhebeimu (*Fritillaria thunbergia* Miq.) 10g;  Shanzha (*Crataegus monogyna* Jacq.) 15g. |
| Chen LQ et al. 2015 |  | *Xuefu Zhuyu* capsules |  | Chishao (*Paeonia lactiflora* Pall.); Taoren (*Prunus*  *persica* (L.) Batsch); Chaihu (*Bupleurum chinense* DC.);  Gancao (*Glycyrrhiza glabra* L.); Danggui (*Angelica*  *sinensis* (Oliv.) Diels); Shudi (*Rehmannia glutinosa*  (Gaertn.) DC.); Chuanxiong (*Conioselinum anthriscoides*  *'Chuanxiong'*); Honghua (*Carthamus tinctorius* L.);  Zhiqiao (*Citrus × aurantium f. aurantium*); Jiegeng  (*Platycodon grandiflorus* (Jacq.) A.DC.); Niuxi  (*Achyranthes bidentata* Blume). |
| Chen JJ 2015 |  | *Bushen Huayu* decoction |  | Chishao (*Paeonia lactiflora* Pall.)15g; Sangjisheng  (*Taxillus chinensis* (DC.) Danser) 20g; Tusizi (*Cuscuta*  *chinensis* Lam.) 15g; Sanleng(*Sparganium stoloniferum*  (Buch.-Ham. ex Graebn.) Buch.-Ham. ex Juz.) 10g; Ezhu  (*Curcuma longa* L.) 10g; Danshen (*Salvia miltiorrhiza*  Bunge) 15g; Yanhusuo (*Corydalis yanhusuo* (Y.H.Chou  & Chun C.Hsu) W.T.Wang ex Z.Y.Su & C.Y.Wu) 15g;  Jixueteng (*Spatholobus suberectus* Dunn) 20g; Qingpi  (*Citrus reticulata* Blanco)10g Gouqi (*Lycium barbarum*  L.) 10g; Xuduan (*Dipsacus asper* Wall. Ex DC.) 10g. |
| Dou N 2015 |  | *Danbie* capsules |  | Guizhi (*Neolitsea cassia* (L.) Kosterm.); Taoren (*Prunus*  *persica* (L.) Batsch); Duzhong (*Eucommia ulmoides*  Oliv.); Sanleng(*Sparganium stoloniferum* (Buch.-Ham.  ex Graebn.) Buch.-Ham. ex Juz.); Ezhu (*Curcuma longa*  L.); Danggui (*Angelica sinensis* (Oliv.) Diels); Danshen  (*Salvia miltiorrhiza* Bunge); Sanqi (*Panax notoginseng*  (Burkill) F.H.Chen); Haizao (*Sargassum pallidum*  (Turn.) C.Ag.); Biejia (*Trionyx sinensis* Wiegmann);  Baizhu (*Atractylodes macrocephala* Koidz.); Banzhilian  (*Scutellaria barbata* D.Don). |
| Li S 2014 |  | Empirical formula |  | Guizhi (*Neolitsea cassia* (L.) Kosterm.) 3g; Fuling  (*Carapichea ipecacuanha* (Brot.) L.Andersson) 20g;  Tusizi (*Cuscuta chinensis* Lam.) 10g; Sanleng(*Sparganium stoloniferum* (Buch.-Ham.  ex Graebn.) Buch.-Ham. ex Juz.) 20g; Ezhu (*Curcuma*  *longa* L.) 20g; Danggui (*Angelica sinensis* (Oliv.) Diels)  15g; Moyao (*Commiphora myrrha* (T.Nees) Engl.) 12g;  Danshen (*Salvia miltiorrhiza* Bunge) 20g; Yanhusuo  (*Corydalis yanhusuo* (Y.H.Chou & Chun C.Hsu)  W.T.Wang ex Z.Y.Su & C.Y.Wu) 12g; Chuanxiong  (*Conioselinum anthriscoides 'Chuanxiong'*) 15g;  Dahuang (*Rheum officinale* Baill.) 6g; Xiaohuixiang  (*Foeniculum vulgare* Mill.) 12g; Huangqi (*Astragalus*  *mongholicus* Bunge) 15g. |
| Zhou D et al. 2013 |  | Empirical formula |  | Mudanpi (*Paeonia × suffruticosa* Andrews) 12g; Chishao  (*Paeonia lactiflora* Pall.) 12g; Taoren (*Prunus persica*  (L.) Batsch) 9g; Yinyanghuo (*Epimedium sagittatum*  (Siebold & Zucc.) Maxim.) 12g; Xiangfu (*Cyperus*  *rotundus* L.)12g; Lizhihe (*Litchi chinensis* Sonn.)12g;  Tusizi (*Cuscuta chinensis* Lam.) 10g; Jineijin (*Gallus*  *gallus domesticus* Brisson) 6g; Danshen (*Salvia*  *miltiorrhiza* Bunge) 30g; Chuanxiong (*Conioselinum*  *anthriscoides 'Chuanxiong'*) 15g; Huangqi (*Astragalus*  *mongholicus* Bunge) 20g; Dangshen (*Codonopsis*  *pilosula* (Franch.) Nannf.) 30g. |
| Zhang XN 2012 |  | *Muda Tang* granules |  | Taoren (*Prunus persica* (L.) Batsch); Chaihu (*Bupleurum*  *chinense* DC.); Yujin (*Curcuma longa* L.); Xiangfu  (*Cyperus rotundus* L.); Gancao (*Glycyrrhiza glabra* L.);  Danggui (*Angelica sinensis* (Oliv.) Diels); Baishao  (*Paeonia lactiflora* Pall.); Yanhusuo (*Corydalis yanhusuo*  (Y.H.Chou & Chun C.Hsu) W.T.Wang ex Z.Y.Su &  C.Y.Wu); Wuyao (*Lindera aggregata* (Sims) Kosterm.);  Zhiqiao (*Citrus × aurantium f. aurantium*). |
| Ding XQ et al. 2012 |  | Empirical formula |  | Guizhi (*Neolitsea cassia* (L.) Kosterm.) 15g; Fuling  (*Carapichea ipecacuanha* (Brot.) L.Andersson) 15g;  Mudanpi (*Paeonia × suffruticosa* Andrews) 15g; Taoren  (*Prunus persica* (L.) Batsch) 10g; Bajitian  (*Gynochthodes officinalis* (F.C.How) Razafim. &  B.Bremer) 15g; Yujin (*Curcuma longa* L.)15g; Xiangfu  (*Cyperus rotundus* L.)15g; Lizhihe (*Litchi chinensis*  Sonn.)15g; Danggui (*Angelica sinensis* (Oliv.) Diels)15g;  Muli (*Ostrea gigas* Thunberg) 15g; Xuduan (*Dipsacus*  *asper* Wall. Ex DC.) 15g; Juhe (*Citrus reticulata* Blanco)  15g; Muxiang (*Dolomiaea costus* (Falc.) Kasana &  A.K.Pandey) 10g; Longgu (Fossilia Ossis Mastodi) 15g;  Lujiaoshuang (*Cervi Cornu Degelatinatum*) 15g. |
| Ma L et al. 2011 |  | *Xiaojie An* capsules |  | Tufuling (*Smilax glabra* Roxb.); Yimucao (*Leonurus*  *japonicus* Houtt.); Jixueteng (*Spatholobus suberectus*  Dunn); Gonglaomu (*Berberis bealei* Fortune); Sanchaku  (*Melicope pteleifolia* (Champ. ex Benth.)  T.G.Hartley); Lianqiao (*Forsythia suspensa* (Thunb.)  Vahl). |
