# Supplementary material for: Exploring the role of Chinese herbal medicine in the long-term management of postoperative ovarian endometriotic cysts: a systematic review and meta-analysis
Source: Front Pharmacol. 2024 Jun 7;15:1376037. doi: 10.3389/fphar.2024.1376037 (PMC11190181; doi:10.3389/fphar.2024.1376037)
Supplement: Supplementary file 3 [file Table2.doc]

# Supplementary Appendix 2

# Statement

All data included in this review were supported by 8 electronic databases for published studies. Appendix-2 shows the search strategies for all the electronic databases.

**Supplement Appendix 2** Search strategies for all the electronic databases

| **Databases** | **Search strategies** | **Time limit** |
| --- | --- | --- |
| CNKI | ((SU=中医 OR SU=中医药 OR SU=中西医 OR SU=中西 OR SU=中药 OR SU=草药 OR SU=成药 OR SU=中成药) OR (SU=汤剂 OR SU=汤 OR SU=丸 OR SU=散 OR SU=膏 OR SU=丹 OR SU=片 OR SU=栓 OR SU=颗粒 OR SU=胶囊 OR SU=滴丸)) AND (SU =卵巢子宫内膜异位囊肿 OR SU =巧囊 OR SU =卵巢巧克力囊肿 OR SU =巧克力囊肿 OR SU %=卵巢型子宫内膜异位症 OR SU %=卵巢型内异症 OR SU %=卵巢EMS OR SU %=卵巢子宫内膜囊肿 OR SU %=子宫内膜囊肿 OR SU %=子宫内膜异位囊肿) AND ( SU %= '术后' OR SU =腹腔镜 OR SU =经腹 OR SU =开腹 OR SU %=手术 OR SU %=保守性手术 OR SU %=剥除OR SU %=切除OR SU %=消融) AND (FT = '随机对照试验' OR FT = '随机') | From inception to November 01, 2023 |
| Wanfang | ( (主题:“中医” or “中药” or “中医药” or “中西医” or “中西” or “中草药” or “中成药” or “草药” or “成药”) or (主题: “丸” or “汤剂” or “散” or “膏” or “丹” or “汤” or “颗粒” or “胶囊” or “滴丸” or “栓”) ) and (主题: “卵巢子宫内膜异位囊肿” or “卵巢子宫内膜异位症” or “巧囊” or “卵巢巧克力囊肿” or “卵巢型子宫内膜异位症” or “卵巢子宫内膜囊肿” or “子宫内膜异位囊肿” or “卵巢EMS” or “卵巢型内异症” ) and (主题: “术后” or “腹腔镜” or “开腹” or “经腹” or “保守性手术” or “剥除”or “切除”or “消融”) and 全部:( “随机对照试验” or “随机”) | From inception to November 01, 2023 |
| VIP | ((M=中医 OR M=中药 OR M=中医药 OR M=中西医 OR M=中西 OR M=中成药 OR M=草药 OR M=成药) OR (M=汤 OR M=丸 OR M=散 OR M=膏 OR M=丹 OR M=片 OR M=栓 OR M=颗粒 OR M=胶囊 OR M=滴丸)) AND ( M=卵巢子宫内膜异位囊肿 OR M=卵巢子宫内膜异位症 OR M=巧囊 OR M=卵巢巧克力囊肿 OR M=卵巢型内异症 OR M=卵巢子宫内膜囊肿 OR M=子宫内膜异位囊肿 OR M=卵巢EMS OR M=卵巢型内异症) AND ( M=术后 OR M=腹腔镜 OR M=开腹 OR M=经腹 OR M=保守性手术 OR M=剥除 OR M=切除 OR M=消融) AND (U=随机对照试验 OR U=随机) | From inception to November 01, 2023 |
| CBM | 1) "中草药"[不加权:扩展]  2) "中药"[常用字段:智能] OR "中医"[常用字段:智能] OR "中医药"[常用字段:智能] OR "中西"[常用字段:智能] OR "中西医"[常用字段:智能] OR "中成药"[常用字段:智能] OR "成药"[常用字段:智能] OR "汤剂"[常用字段:智能] OR "汤"[常用字段:智能]  3) "汤"[常用字段:智能] OR "丸"[常用字段:智能] OR "散"[常用字段:智能] OR "膏"[常用字段:智能] OR "丹"[常用字段:智能] OR "胶囊"[常用字段:智能] OR "滴丸"[常用字段:智能] OR "颗粒"[常用字段:智能] OR "栓"[常用字段:智能]  4) "卵巢子宫内膜异位囊肿"[常用字段:智能] OR "卵巢子宫内膜异位症"[常用字段:智能] OR "卵巢型内异症"[常用字段:智能] OR "卵巢子宫内膜囊肿"[常用字段:智能] OR "卵巢巧克力样囊肿"[常用字段:智能] OR "巧克力囊肿"[常用字段:智能] OR "巧囊"[常用字段:智能] OR "子宫内膜囊肿"[常用字段:智能]  5) "手术后期间"[不加权:扩展]  6) "术后"[常用字段:智能]  7) "腹腔镜检查"[不加权:扩展]  8) "经腹"[常用字段:智能] OR "开腹"[常用字段:智能] OR "剥除"[常用字段:智能] OR "切除"[常用字段:智能] OR "消融"[常用字段:智能]  9) (#3) OR (#2) OR (#1)  10) (#8) OR (#7) OR (#6) OR (#5)  11) (#10) AND (#9) AND (#4)  12) "随机对照试验"[不加权:扩展]  13) 随机  14) (#13) OR (#12)  15) (#14) AND (#11) | From inception to November 01, 2023 |
| PubMed | (((((((((((((("Medicine, Chinese Traditional"[Mesh]) OR (((((Traditional Chinese Medicine) OR (Traditional Medicine, Chinese)) OR (Zhong Yi Xue)) OR (Chinese Traditional Medicine)) OR (Chinese Medicine, Traditional)))) OR (formula)) OR (decoction)) OR (recipe)) OR (pill)) OR (powder)) OR (paste)) OR (pellet)) OR (tablet)) OR (granules)) OR ((capsule) OR (extract))) AND (((Ovarian endometriotic cyst[MeSH Terms]) OR (((Ovarian endometriosis) OR (Ovarian endometrioma*)) OR (Ovarian chocolate cyst*))))) AND (((((((Surgical Procedures, Operative[MeSH Terms]) OR (((((((Operative Procedure*) OR (Procedure* Operative)) OR (Surgical Procedure, Operative)) OR (Operative Surgical Procedure*)) OR (Procedure*, Operative Surgical)) OR (Surgical Procedure*)) OR (Procedure*, Surgical))) OR ((Laparoscopes[MeSH Terms]) OR ((Peritoneoscope*) OR (Celioscope*)))) OR ((ablation techniques[MeSH Terms]) OR ((Ablation Technique*) OR (Technique*, Ablation)))) OR (excision)) OR ((Postoperative Period[MeSH Terms]) OR (((Postoperative Period*) OR (Period*, Postoperative)) OR (Postoperative))))) Filters: Randomized Controlled Trial | From inception to November 01, 2023 |
| Cochrane Library | #1 MeSH descriptor: [Medicine, Chinese Traditional] explode all trees  #2 (Traditional Chinese Medicine):ti,ab,kw OR (Traditional Medicine, Chinese):ti,ab,kw OR (Zhong Yi Xue):ti,ab,kw OR (Chinese Traditional Medicine):ti,ab,kw OR (Chinese Medicine, Traditional):ti,ab,kw OR (formula):ti,ab,kw OR (decoction) :ti,ab,kw OR (recipe):ti,ab,kw OR (pill):ti,ab,kw OR (powder):ti,ab,kw OR (paste):ti,ab,kw OR (pellet):ti,ab,kw OR (tablet):ti,ab,kw OR (granules):ti,ab,kw OR (capsule):ti,ab,kw OR (extract):ti,ab,kw (Word variations have been searched)  #3 #1 or #2  #4 (Ovarian endometriotic cyst):ti,ab,kw OR (Ovarian endometriosis):ti,ab,kw OR (Ovarian endometrioma*):ti,ab,kw OR (Ovarian chocolate cyst*):ti,ab,kw  #5 MeSH descriptor: [Surgical Procedures, Operative] explode all trees  #6 (Operative Procedure*):ti,ab,kw OR (Procedure* Operative):ti,ab,kw OR (Surgical Procedure, Operative):ti,ab,kw OR (Operative Surgical Procedure*):ti,ab,kw OR (Procedure*, Operative Surgical):ti,ab,kw (Word variations have been searched)  #7 (Surgical Procedure*):ti,ab,kw OR (Procedure*, Surgical):ti,ab,kw (Word variations have been searched)  #8 #5 or #6 or #7  #9 MeSH descriptor: [Laparoscopes] explode all trees  #10 (Peritoneoscope*):ti,ab,kw OR (Celioscope*):ti,ab,kw (Word variations have been searched)  #11 #9 or #10  #12 MeSH descriptor: [Ablation Techniques] explode all trees  #13 (Ablation Technique):ti,ab,kw OR (Technique*, Ablation):ti,ab,kw  #14 #12 or #13  #15 (excision):ti,ab,kw  #16 MeSH descriptor: [Postoperative Period] explode all trees  #17 (Postoperative Period*):ti,ab,kw OR (Period*, Postoperative):ti,ab,kw OR (Postoperative):ti,ab,kw  #18 #16 or #17  #19 #8 or #11 or #14 or #15 or #18  #20 #3 and #4 and #19 | From inception to November 01, 2023 |
| Embase | #46. #18 AND #22 AND #45  #45. #31 OR #35 OR #39 OR #40 OR #44  #44. #41 OR #42 OR #43  #43. 'postoperative':ab,ti  #42. 'period*, postoperative':ab,ti  #41. 'postoperative period'/exp  #40. 'excision'/exp  #39. #36 OR #37 OR #38  #38. 'technique*, ablation':ab,ti  #37. 'ablation technique*':ab,ti  #36. 'ablation therapy'/exp  #35. #32 OR #33 OR #34  #34. 'celioscope*':ab,ti  #33. 'peritoneoscope*':ab,ti  #32. 'laparoscope'/exp  #31. #23 OR #24 OR #25 OR #26 OR #27 OR #28 OR #29 OR #30  #30. 'procedure*, surgical':ab,ti  #29. 'surgical procedure*':ab,ti  #28. 'procedure*, operative surgical':ab,ti  #27. 'operative surgical procedure*':ab,ti  #26. 'surgical procedure, operative':ab,ti  #25. 'procedure* operative':ab,ti  #24. 'operative procedure*':ab,ti  #23. 'surgery'/exp  #22. #19 OR #20 OR #21  #21. 'ovarian endometrioma*':ab,ti OR 'ovarian  chocolate cyst*':ab,ti  #20. 'ovarian endometriosis':ab,ti  #19. 'ovarian endometriotic cyst'/exp  #18. #1 OR #2 OR #3 OR #4 OR #5 OR #6 OR #7 OR #8 OR #9 OR #10 OR #11 OR #12 OR #13 OR #14 OR #15 OR #16 OR #17  #17 'extract':ti,ab  #16 'capsule':ti,ab  #15 'granules':ti,ab  #14 'tablet':ti,ab  #13 'pellet':ti,ab  #12 'paste':ti,ab  #11 'powder':ti,ab  #10 'pill':ti,ab  #9 'recipe':ti,ab  #8 'decoction ':ti,ab  #7 'formula':ti,ab  #6. 'chinese medicine, traditional':ti,ab  #5. 'chinese traditional medicine':ti,ab  #4. 'zhong yi xue':ti,ab  #3. 'traditional medicine, chinese':ti,ab  #2. 'traditional chinese medicine':ti,ab  #1. 'chinese medicine'/exp | From inception to November 01, 2023 |
| Web of Science | # 4  #3 AND #2 AND #1  # 3  TS=(Surgical Procedures OR Operative Procedure* OR Procedure* Operative OR Surgical Procedure, Operative OR Operative Surgical Procedure* OR Procedure*, Operative Surgical OR Surgical Procedure* OR Procedure*, Surgical OR Laparoscopes OR Peritoneoscope* OR Celioscope* OR Ablation Technique* OR Technique*, Ablation OR excision OR Postoperative Period* OR Period*, Postoperative OR Postoperative)  # 2  TS=(Ovarian endometriotic cyst OR Ovarian endometriosis OR Ovarian endometrioma* OR Ovarian chocolate cyst*)  # 1  TS=(Medicine, Chinese Traditional OR Traditional Chinese Medicine OR Traditional Medicine, Chinese OR Zhong Yi Xue OR Chinese Traditional Medicine OR Chinese Medicine, Traditional OR formula OR decoction OR recipe OR pill OR powder OR paste OR pellet OR tablet OR granules OR capsule OR extract) | From inception to November 01, 2023 |
